# Comparing similarities and differences between NAFLD, MAFLD, and MASLD in the general U.S. population

**DOI:** 10.3389/fnut.2024.1411802

**Published:** 2024-07-08

**Authors:** Haoxuan Zou, Xiaopu Ma, Wen Pan, Yan Xie

**Affiliations:** ^1^Department of Gastroenterology and Hepatology, West China Hospital, Sichuan University, Chengdu, Sichuan, China; ^2^Department of Health Management Center, The Hospital of Chengdu Office of People's Government of Tibetan Autonomous Region, Chengdu, Sichuan, China

**Keywords:** SLD, NAFLD, MAFLD, MASLD, NHANES

## Abstract

**Background:**

Recently, the multisociety Delphi consensus renamed non-alcoholic fatty liver disease (NAFLD) terminology [previously renamed metabolic-associated fatty liver disease (MAFLD)] as metabolic dysfunction-associated steatotic liver disease (MASLD). The aim of this study was to compare the similarities and differences between NAFLD, MAFLD, and MASLD and to clarify the impact of this new name change.

**Methods:**

A cross-sectional study of 3,035 general subjects with valid vibration-controlled transient elastography data was conducted based on data from the National Health and Nutrition Examination Survey (NHANES) 2017–2020. NAFLD, MAFLD, and MASLD were defined according to the corresponding consensus criteria.

**Results:**

Using controlled attenuation parameter (CAP) ≥274 dB/m and liver stiffness measurements (LSM) ≥9.7 kPa as the cutoff values for the presence of hepatic steatosis and advanced liver fibrosis (ALF), the prevalence of NAFLD, MAFLD, and MASLD were 38.01% (95% CI 35.78–40.29%), 41.09% (39.09–43.12%), and 37.9% (35.70–40.14%), respectively, and the corresponding prevalence of ALF was 10.21% (7.09–14.48%), 10.13% (7.06-14.35%), and 10.24% (7.11–14.53%), respectively. The kappa values for the three definitions were above 0.9. The prevalence and severity of the three definitions remained similar when the sensitivity analyses were performed using different CAP thresholds. The prevalence of NAFLD, MAFLD, MASLD, and ALF increased as the number of cardiometabolic risk factors (CMRF) increased.

**Conclusions:**

Our findings highlight the consistency among the three definitions, especially between NAFLD and MASLD, so that the new consensus will not disturb the original NAFLD-related findings. Additionally, more attention should be paid to patients with a high number of CMRFs.

## Introduction

Non-alcoholic fatty liver disease (NAFLD) has emerged as the most common chronic liver disease worldwide, with an estimated prevalence exceeding one-third of the population (1, 2). NAFLD encompasses a spectrum of diseases, including non-alcoholic fatty liver (NAFL) and non-alcoholic steatohepatitis (NASH), as well as its consequential cirrhosis and hepatocellular carcinoma (HCC) (3, 4). However, the exclusionary diagnostic criteria for NAFLD have gradually revealed limitations in their long-term clinical application. These criteria fail to adequately account for the diverse etiological origins of fatty liver disease and do not support comprehensive and effective treatment of patients with multiple chronic liver diseases.

In 2020, a group of 31 experts from 22 countries, proposed the substitution of NAFLD with the term metabolic dysfunction-associated fatty liver disease (MAFLD). This new nomenclature was accompanied by the establishment of affirmative diagnostic criteria (5). The adoption of MAFLD nomenclature serves to underscore the fundamental role of metabolic factors in the etiology of hepatic fat accumulation in this particular liver disease. Furthermore, this is the first time that the clinical significance of metabolic factor-induced liver lesions has been elevated to a level equivalent to that of other chronic liver diseases, such as viral hepatitis and alcoholic liver disease. Nevertheless, the current MAFLD nomenclature retains the potentially stigmatizing term “fatty” while lacking a precise delineation of the clinical categorization for fatty liver disease and alcohol consumption, thereby giving rise to heightened apprehensions regarding the amalgamation of etiologies (6).

Three years later, the Delphi consensus statement, led by the American Association for the Study of Liver Diseases (AASLD), the European Association for the Study of the Liver (EASL), and the Asociación Latinoamericana parael Estudio del Hígado (ALEH) and co-authored by 53 experts from around the globe, proposed adopting the term “steatotic liver disease” (SLD) as an integrative description of the various etiologies of steatosis. In addition, they proposed replacing “NAFLD” with “metabolic dysfunction-associated steatotic liver disease (MASLD),” which provided an inclusive, non-misleading description of the disease (7).

A limited number of studies have conducted preliminary investigations into the prevalence of SLDs and their subclassifications. Ciardullo et al. utilized data from the 2017 to 2020 cycle of the National Health and Nutrition Examination Survey (NHANES) to diagnose SLD (with a cutoff of controlled attenuation parameter (CAP) ≥274 dB/m) using vibration-controlled transient elastography (VCTE). Their findings revealed that the prevalence of SLD in the United States population was 42.1% (95% CI 40.3–43.9%). Among those diagnosed with SLD, 89.4% exhibited MASLD, 7.7% had MASLD and increased alcohol intake (MetALD), 2.4% had MASLD with viral hepatitis, 0.4% had ALD, and 0.1% had cryptogenic SLD (8). Lee et al. similarly applied the NHANES and used CAP ≥ 285 dB/m as the cutoff for the diagnosis of SLD; the prevalence of SLD and its subcategories was SLD 34.2% (95% CI 31.9–36.5%), MASLD 31.3% (29.2–33.4%), MetALD 2.8% (2.2–3.6%), ALD 0.07% (0.02–0.18%), and etiology-specific/cryptogenic 0.03% (0.01–0.08%) (9). As MASLD accounts for the vast majority of SLDs, the clinical features, prevalence, and severity of the three nomenclatures and diagnoses (NAFLD, MAFLD, and MASLD) deserve further exploration to clarify the value and impact of these nomenclature changes.

The objective of this study was to investigate the prevalence, severity, and characterization of NAFLD/MAFLD/MASLD using CAP and liver stiffness measurement (LSM) data derived from the latest NHANES spanning from 2017 to March 2020, with the aim of examining the influence of the updated nomenclature.

## Materials and methods

### Data source

This study utilized data from the National Health and Nutrition Examination Survey (NHANES) conducted from 2017 to 2020.3. NHANES is a comprehensive cross-sectional study that encompasses a diverse array of population characteristics, health status, nutritional intake, physical measurements, and laboratory tests. It holds national representation and is extensively employed in research, policy formulation, and public health decision making. All participants were required to complete consent forms before engaging in the study, and the survey protocol received approval from the ethics review board of the National Center for Health Statistics (NCHS) Research.

### Demographic, anthropometric, laboratory, lifestyle, and comorbidity data

The NHANES was utilized as the primary data source and included various variables such as demographic parameters, anthropometric parameters, lifestyles, comorbidities, and laboratory factors. Further elaboration of these variables can be found in the Supplementary material. Supplementary material also provide comprehensive definitions of demographics, lifestyle factors, and comorbidities including race, smoking status (10), alcohol consumption (11), viral hepatitis (12), diabetes (13), and hypertension (14).

### Definition of SLD, subclassification of SLD, NAFLD, MAFLD, ALF, and progressive NASH

In identifying hepatic steatosis and fibrosis in individuals, CAP and liver stiffness measurement (LSM) through VCTE have proven to be effective (15, 16). In this specific study, we utilized a cutoff of CAP ≥274 dB/m to indicate the presence of significant hepatic steatosis, which provided an AUC of 0.87 (0.82–0.92), a sensitivity (SEN) of 0.90 (0.87–0.93), and a specificity (SPE) of 0.66 (0.61–0.71) according to Eddowe et al.'s study (17). Sensitivity analyses were conducted for cutoffs of CAP ≥248 dB/m [AUC 0.82 (0.81–0.84), SEN 0.69 (0.60–0.75), and SPE 0.82 (0.76–0.90)] (18), as well as CAP ≥302 dB/m [AUC 0.87 (0.82–0.92), SEN 0.80 (0.75–0.84), and SPE 0.83 (0.69–0.92)] to evaluate the impact of different cutoff values (17). In the context of this study, the participants were categorized according to the LSM into specific stages of hepatic fibrosis, specifically, hepatic fibrosis stage 2 or higher (LSM ≥8.2 kPa), hepatic fibrosis stage 3 or higher (LSM ≥9.7 kPa), and hepatic fibrosis stage 4 (LSM ≥13.6 kPa), with the definition of ALF being an LSM ≥9.7 kPa (17). To identify progressive NASH, we used the FibroScan aminotransferase (FAST) score, the formula for which is detailed in the Supplementary material, and a FAST score ≥0.67 indicated the presence of progressive NASH (19).

The presence of significant hepatic steatosis was determined as SLD based on the most recent Delphi consensus (7). SLD was further classified into five subclasses, namely, MASLD, MetALD, ALD, MASLD-viral, and cryptogenic SLD. MASLD was defined as the presence of significant hepatic steatosis and one or more cardiometabolic risk factors (CMRF), whereas patients with excessive alcohol consumption (>140 grams per week for women and >210 grams per week for men) and other causes of hepatic steatosis were excluded. NAFLD is defined by the presence of significant hepatic steatosis and the absence of excessive alcohol consumption and other causes of hepatic steatosis. MAFLD was defined as the presence of hepatic steatosis with any of the following conditions: overweight/obesity, diabetes, or metabolic dysfunction (20, 21) (detailed definitions of these classifications can be found in the Supplementary material).

### Statistical analysis

To ensure the representativeness of our findings, we adhered to the NHANES analytical guidelines and utilized the 2-year sample weights provided by the NCHS-edited analysis guide (22). Continuous variables were presented as the mean [standard error of the mean (SE)], and the *P*-value was determined using the weighted linear regression model. Categorical values were expressed as % (SE), and *P*-value were computed using the weighted chi-square test. The incorporation of sample weights was considered in the calculations of all estimates.

Weighted logistic regression models were constructed to analyze the association between NAFLD/MAFLD/MASLD and ALF/progressive NASH. As potential confounders, covariates were included if they were significantly associated with ALF/progressive NASH according to logistic regression analysis (*P* < 0.05) or if the inclusion of covariates changed the effect size by >10% or based on clinical experience.

A significance level of 0.05 was employed for all statistical tests, and the analyses were conducted using R software (version 4.2.2).

## Results

### Study population

**Figure 1:**
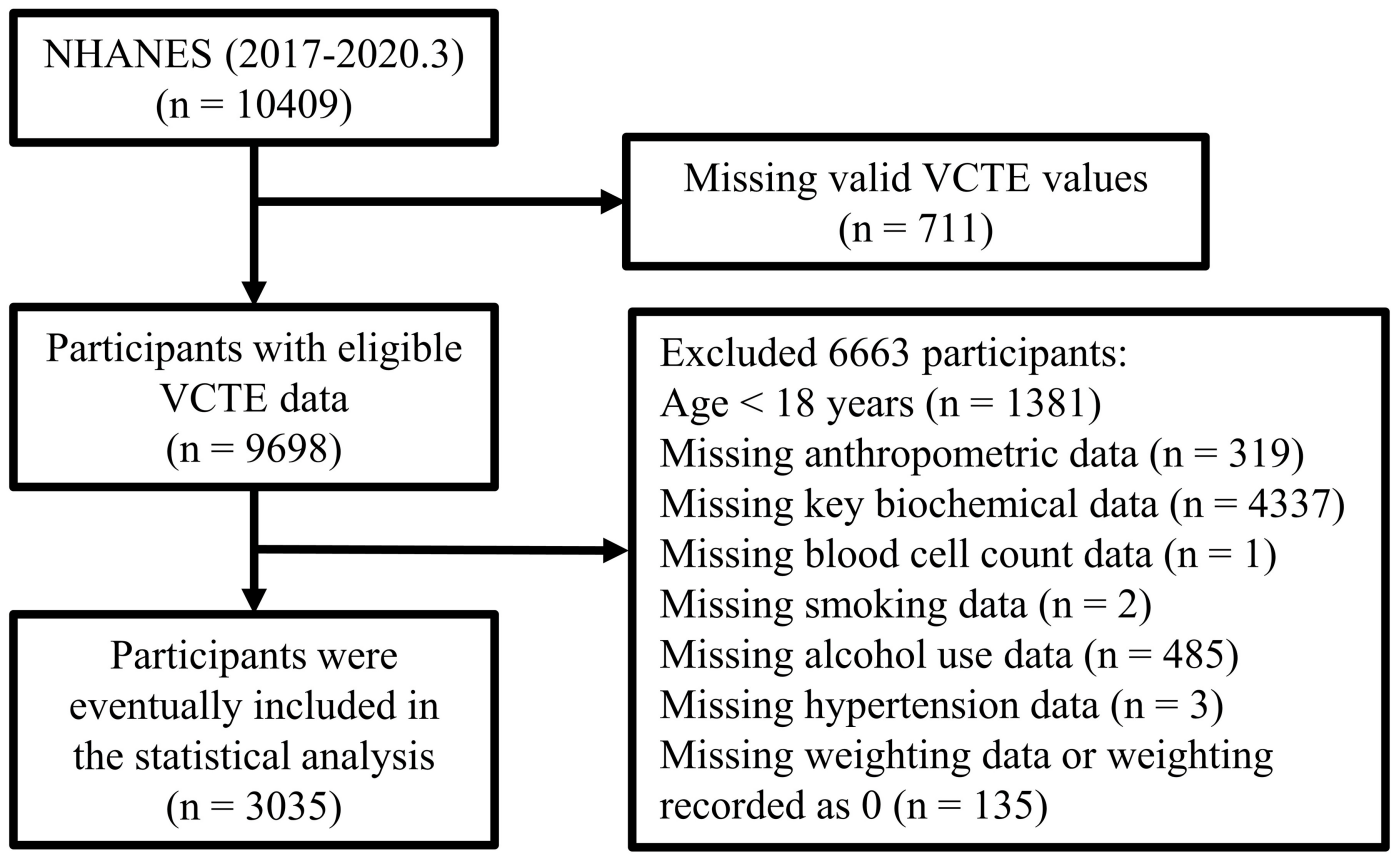
Flow chart of the study participants.

Overall, 10,409 subjects were enrolled in the NHANES database for the 2017–2030.3 survey. As shown in Figure 1, after conducting stepwise exclusions for various reasons, such as lack of valid VCTE values (*n* = 711), age younger than 18 years (*n* = 1,381), absence of key anthropometric data (*n* = 319), missing biochemical or blood cell count values (*n* = 4,338), lack of smoking or alcohol use data (*n* = 487), missing key comorbidity records (*n* = 3), and lack of weighting data or weighting recorded as 0 (*n* = 135), a total of 3,035 subjects were ultimately included in the final analyses.

The weighted prevalence of SLD was 42.42% (95% CI: 40.52–44.34%), and the weighted prevalence of its subclassifications were 37.90% (35.70–40.14%) for MASLD, 3.33% (2.61–4.24%) for MetALD, 0.19% (0.07–0.27%) for ALD, 0.88% (0.19–3.93%) for MASLD-viral, and 0.11% (0.05–0.27%) for cryptogenic SLD. The clinical characteristics of the SLD subclassifications are shown in Supplementary Table 1, and patients with MASLD accounted for the majority of the SLD (89.79%). In the context of sensitivity analyses involving various CAP thresholds, it was observed that patients with MASLD continued to account for the vast majority of SLDs (CAP ≥248 dB/m: 87.65%, CAP ≥302 dB/m: 91.50%).

### Assessment of similarities and differences between NAFLD, MAFLD, and MASLD

In comparison to non-MASLD participants, as shown in Table 1, MASLD patients were more advanced in age; had a greater proportion of male and Hispanic individuals; had an elevated body mass index (BMI) and waist circumference (WC); had increased fasting plasma glucose (FPG), triglyceride (TG), glycated hemoglobin (Hb1Ac), homeostatic model assessment of insulin resistance (HOMA-IR), uric acid (UA), high-sensitivity C-reactive protein (HSCRP), alanine aminotransferase (ALT), gamma-glutamyl transferase (GGT), and alkaline phosphatase (ALP) levels; and had a greater proportion of each fibrosis stage (all *P* < 0.05). Conversely, MASLD patients had lower percentages of albumin (ALB), high-density lipoprotein (HDL), and non-Hispanic Black individuals (all *P* < 0.05). No statistically significant differences were observed in platelet (PLT) counts or creatinine (CRE) levels. Similar findings were also noted in the NAFLD and MAFLD cohorts compared to the non-NAFLD and non-MAFLD cohorts, respectively.

**Table 1:** Demographic, clinical, and laboratory parameters of NAFLD, MAFLD, and MASLD in NHANES 2017–2020.3.

**Variable**	**Non-NAFLD**	**NAFLD**	**Non-MAFLD**	**MAFLD**	**Non-MASLD**	**MASLD**
*N* (weighted prevalence)	1,824 (61.99)	1,211 (38.01)	1,743 (58.91)	1,292 (41.09)	1,830 (62.10)	1,205 (37.90)
Age (years)	44.85 (0.79)	50.61 (0.84)^a^	44.35 (0.86)	50.89 (0.81)^a^	44.83 (0.78)	50.67 (0.84)^a^
Male (%)	47.77 (1.57)	56.71 (2.49)^a^	47.46 (1.60)	56.47 (2.21)^a^	47.80 (1.59)	56.67 (2.48)^a^
**Race (%)**						
Non-Hispanic black	11.81 (1.45)	8.89 (1.36)^a^	11.99 (1.47)	8.84 (1.37)^a^	11.86 (1.43)	8.79 (1.38)^a^
Non-Hispanic white	65.02 (1.94)	62.83 (2.56)	64.78 (2.14)	63.34 (2.36)	64.94 (1.93)	62.96 (2.58)
Hispanic	13.93 (1.46)	19.66 (2.12)^a^	13.94 (1.46)	19.22 (2.09)^a^	13.96 (1.46)	19.63 (2.14)^a^
Non-Hispanic Asian	4.60 (0.81)	4.07 (0.79)	4.69 (0.84)	3.98 (0.79)	4.60 (0.80)	4.08 (0.80)
Other	4.64 (0.64)	4.56 (1.00)	4.60 (0.62)	4.61 (0.98)	4.64 (0.64)	4.54 (1.00)
WC (cm)	92.98 (0.52)	111.55 (0.79)^a^	91.99 (0.55)	111.56 (0.70)^a^	92.94 (0.52)	111.65 (0.80)^a^
BMI (kg/m^2^)	26.76 (0.22)	34.04 (0.31)^a^	26.46 (0.23)	33.92 (0.29)^a^	26.75 (0.22)	34.08 (0.31)^a^
PLT (10^9^/L)	241.36 (2.27)	242.67 (3.00)	240.92 (2.25)	243.21 (2.53)	241.31 (2.27)	242.75 (3.00)
TBIL (mg/dL)	0.52 (0.02)	0.49 (0.01)	0.53 (0.02)	0.48 (0.01)	0.52 (0.02)	0.49 (0.01)
ALT (U/L)	20.55 (0.55)	25.96 (0.75)^a^	19.76 (0.51)	26.68 (0.75)^a^	20.53 (0.55)	26.00 (0.76)^a^
AST (U/L)	21.57 (0.48)	21.84 (0.47)	20.90 (0.39)	22.77 (0.66)^a^	21.56 (0.48)	21.85 (0.48)
GGT (U/L)	25.70 (0.71)	32.93 (1.23)^a^	24.04 (0.69)	34.77 (1.16)^a^	25.68 (0.71)	32.99 (1.24)^a^
ALP (U/L)	72.48 (0.97)	77.54 (0.97)^a^	71.72 (0.86)	78.24 (0.95)^a^	72.50 (0.96)	77.51 (0.97)^a^
ALB (g/dL)	41.26 (0.16)	40.09 (0.19)^a^	41.30 (0.16)	40.11 (0.19)^a^	41.25 (0.16)	40.09 (0.19)^a^
HbA1C (%)	5.46 (0.02)	5.95 (0.05)^a^	5.44 (0.02)	5.93 (0.06)^a^	5.46 (0.02)	5.96 (0.05)^a^
FPG (mg/dL)	95.30 (0.56)	111.67 (1.77)^a^	94.73 (0.53)	111.26 (1.72)^a^	95.29 (0.56)	111.74 (1.77)^a^
TG (mg/dL)	105.02 (1.90)	154.47 (4.81)^a^	100.80 (1.40)	156.81 (4.80)^a^	104.98 (1.90)	154.68 (4.84)^a^
TC (mg/dL)	184.04 (1.55)	186.38 (2.65)	182.83 (1.59)	187.94 (2.57)	183.99 (1.56)	186.47 (2.65)
HDL (mg/dL)	57.51 (0.59)	48.17 (0.73)^a^	57.68 (0.62)	48.62 (0.83)^a^	57.50 (0.59)	48.16 (0.73)^a^
FINS (μU/mL)	9.61 (0.33)	19.86 (1.01)^a^	9.22 (0.31)	19.65 (0.93)^a^	9.60 (0.33)	19.91 (1.01)^a^
HOMA-IR	2.59 (0.12)	6.16 (0.40)^a^	2.47 (0.11)	6.06 (0.36)^a^	2.59 (0.12)	6.17 (0.40)^a^
CRE (mg/dL)	0.86 (0.01)	0.87 (0.01)	0.86 (0.01)	0.87 (0.01)	0.86 (0.01)	0.87 (0.01)
UA (mg/dL)	5.18 (0.06)	5.84 (0.04)^a^	5.11 (0.05)	5.88 (0.04)^a^	5.18 (0.06)	5.84 (0.04)^a^
HSCRP (mg/L)	3.16 (0.27)	4.80 (0.25)^a^	3.04 (0.27)	4.85 (0.21)^a^	3.15 (0.26)	4.81 (0.25)^a^
CAP (dB/m)	227.36 (1.24)	323.81 (1.83)^a^	222.10 (1.29)	324.13 (1.73)^a^	227.49 (1.22)	323.89 (1.84)^a^
LSM (kPa)	5.17 (0.08)	6.67 (0.27)^a^	5.08 (0.08)	6.69 (0.26)^a^	5.17 (0.08)	6.67 (0.28)^a^
FAST	0.08 (0.00)	0.17 (0.01)^a^	0.07 (0.00)	0.18 (0.01)^a^	0.08 (0.00)	0.17 (0.01)^a^
**Smoking (%)**						
Never	53.86 (1.72)	55.57 (2.24)	56.08 (1.98)	52.27 (2.21)	53.88 (1.73)	55.55 (2.25)
Former	26.40 (1.54)	31.80 (2.19)	26.31 (1.52)	31.53 (1.87)	26.42 (1.56)	31.78 (2.20)
Current	19.74 (1.50)	12.63 (1.74)^a^	17.61 (1.78)	16.21 (1.77)	19.70 (1.51)	12.67 (1.74)^a^
Viral hepatitis (%)	3.61 (1.14)	0.00 (0.00)^a^	2.33 (0.59)	2.11 (1.57)	3.60 (1.14)	0.00 (0.00)^a^
Excessive alcohol intake (%)	12.86 (1.36)	0.00 (0.00)^a^	8.46 (1.38)	7.28 (0.77)	12.84 (1.36)	0.00 (0.00)^a^
Hypertension (%)	23.49 (1.48)	43.34 (2.70)^a^	22.78 (1.53)	42.87 (2.97)^a^	23.44 (1.48)	43.47 (2.73)^a^
Diabetes (%)	8.35(0.77)	26.93 (1.71)^a^	7.80 (0.80)	26.33 (1.87)^a^	8.33 (0.77)	27.01 (1.72)^a^
FAST ≥ 0.67(%)	0.91 (0.44)	3.51 (0.70)^a^	0.67 (0.45)	3.66 (0.70)^a^	0.91 (0.44)	3.52 (0.71)^a^
≥F2 (%)	4.36 (0.59)	14.94 (1.79)^a^	3.82 (0.52)	14.92 (1.87)^a^	4.35 (0.59)	14.99 (1.80)^a^
≥F3 (%)	2.71 (0.61)	10.21 (1.77)^a^	2.37 (0.60)	10.13 (1.75)^a^	2.70 (0.61)	10.24 (1.78)^a^
F4 (%)	1.28 (0.31)	4.62 (0.98)^a^	1.09 (0.35)	4.65 (0.90)^a^	1.28 (0.31)	4.63 (0.98)^a^

Continuous variables are shown as mean [standard error of mean (SE)]. Categorical values are shown as % (SE).

WC, waist circumference; BMI, body mass index; PLT, platelets; TBIL, total bilirubin; ALT, alanine aminotransferase; AST, aspartate aminotransferase; GGT, γ-glutamyl transpeptidase; ALP, alkaline phosphatase; ALB, albumin; HbA1c, hemoglobin A1c; FPG, fasting plasma glucose; TC, total cholesterol; TG, triglyceride; HDL, high-density lipoprotein cholesterol; FINS, fasting insulin; HOMA-IR, homeostasis model assessment of insulin resistance; UA, uric acid; CRE, creatinine; HSCRP, hypersensitive C reactive protein; CAP, controlled attenuation parameter; LSM, liver stiffness measurements; FAST, FibroScan-AST; F2, stage 2 fibrosis; F3, stage 3 fibrosis; F4, stage 4 fibrosis.

^a^Significantly different from controls (P < 0.05).

**Figure 2:**
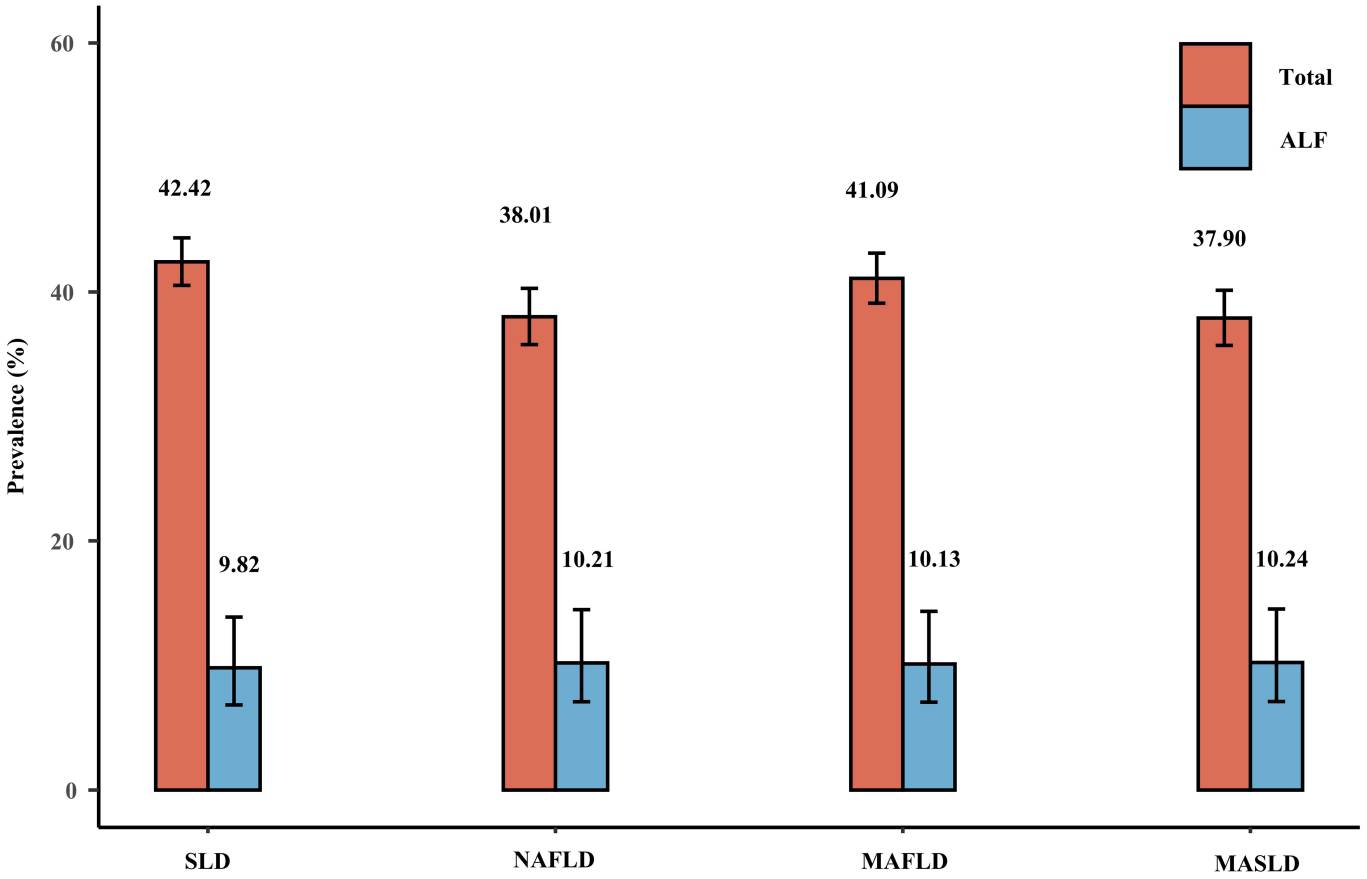
Weighted prevalence of SLD (CAP ≥274 dB/m), NAFLD, MAFLD, and MASLD and associated prevalence of ALF.

Figure 2 shows the weighted prevalence of NAFLD, MAFLD, and MASLD in the overall population: 38.01% (35.78–40.29%) for NAFLD, 41.09% (39.09–43.12%) for MAFLD, and 37.90% (35.70–40.14%) for MASLD. The concordance among the three definitions was high, as evidenced by the kappa values of 0.919 for NAFLD and MAFLD, 0.996 for NAFLD and MASLD, and 0.923 for MAFLD and MASLD. The prevalence of NAFLD, MAFLD, and MASLD corresponding to CAP cutoff values of ≥248 and ≥302 dB/m were also very similar, as shown in Supplementary Figure 1 and Supplementary Tables 2, 4. Within the cohort of patients diagnosed with NAFLD, 2.04% were not classified as MAFLD, whereas 0.30% were not categorized as MASLD. Notably, all of the individuals who did not meet the criteria for MASLD were instead classified as having cryptogenic SLD. Furthermore, it is worth mentioning that only a subset of these non-MAFLD NAFLD patients presented with single prediabetes or elevated HSCRP or lowered HDL, which consequently led to their exclusion from the diagnostic criteria for MAFLD. In the group of patients diagnosed with MASLD, all of whom met the NAFLD diagnostic criteria but 1.74% were not classified as MAFLD, these non-MAFLD MASLD patients, like the non-MAFLD NAFLD patients, were excluded from the MAFLD diagnostic criteria because all of the patients had a normal body weight, did not have diabetes, and only partially met a single metabolic risk abnormality and therefore did not meet the diagnostic criteria for MAFLD. In contrast, 9.37% of the group of patients diagnosed with MAFLD were not categorized as NAFLD/MASLD, and all of these patients had excessive alcohol consumption or HCV infection (the above percentages are weighted).

**Figure 3:**
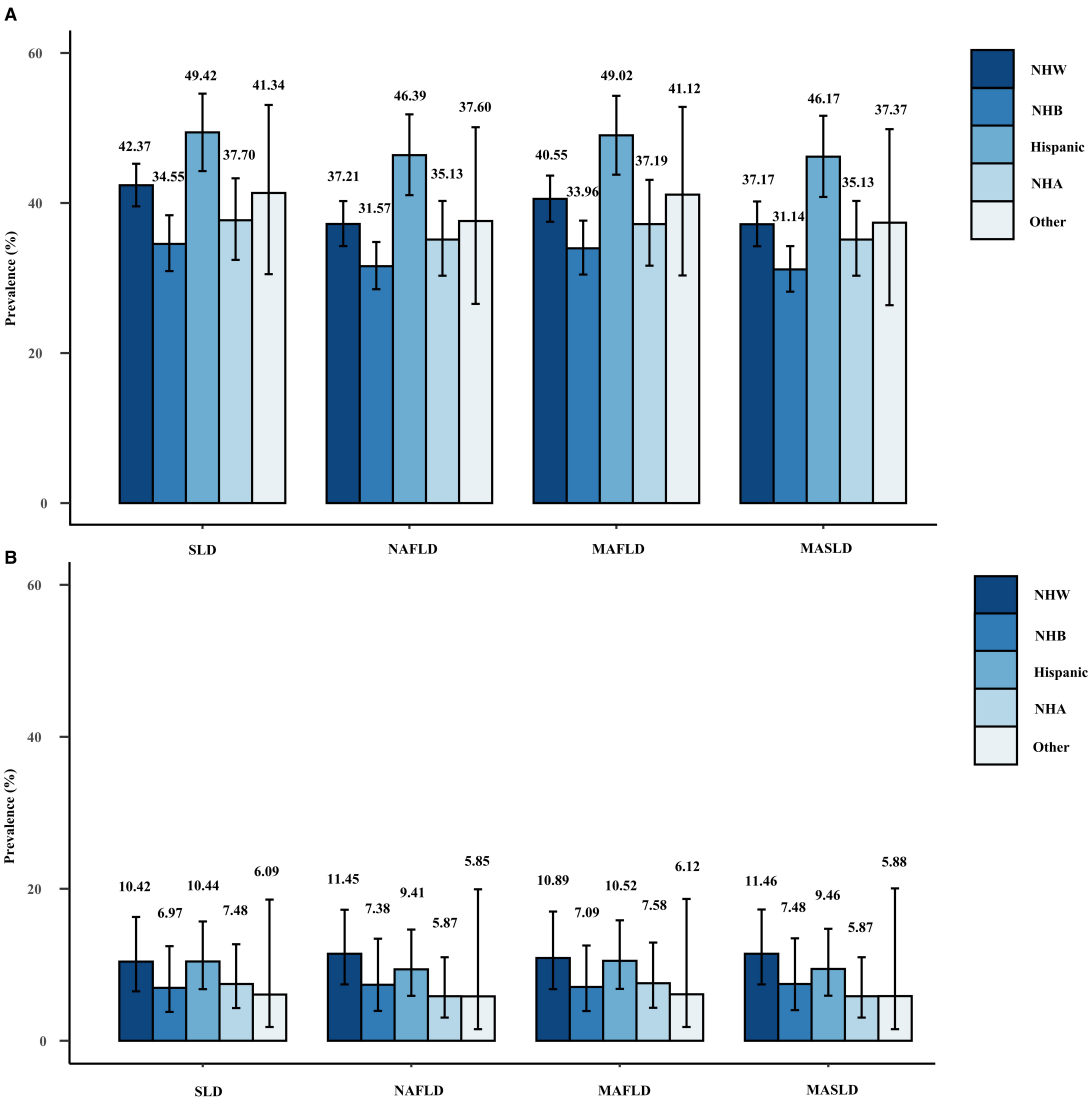
Weighted prevalence of SLD (CAP ≥274 dB/m), NAFLD, MAFLD, and MASLD **(A)** and associated prevalence of ALF **(B)** in different races.

Figure 3 shows the weighted prevalence rates of MAFLD, MASLD, and NAFLD, and the corresponding weighted prevalence rates of ALF across racial groups. The prevalence of any of the disease definitions was highest among Hispanic participants and lowest among non-Hispanic blacks, whereas the corresponding prevalence of ALF was highest among non-Hispanic whites, followed by Hispanics. The aforementioned statement holds true for the outcomes derived from the sensitivity analyses performed with varying CAP thresholds (Supplementary Figures 2, 3).

### Metabolic risk abnormalities in NAFLD/MAFLD/MASLD

**Figure 4:**
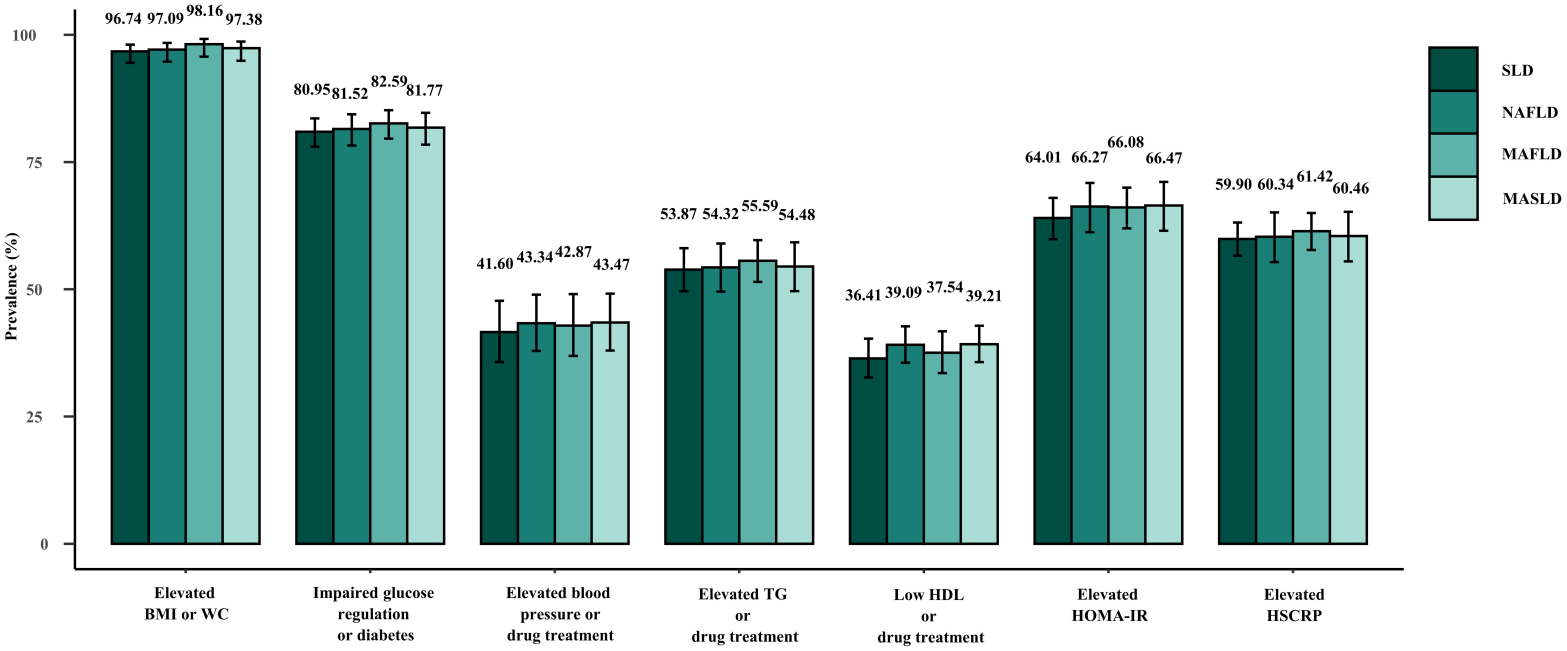
Weighted prevalence of each constituent of the CMRF definition among patients with SLD (CAP ≥274 dB/m), NAFLD, MAFLD, and MASLD.

**Figure 5:**
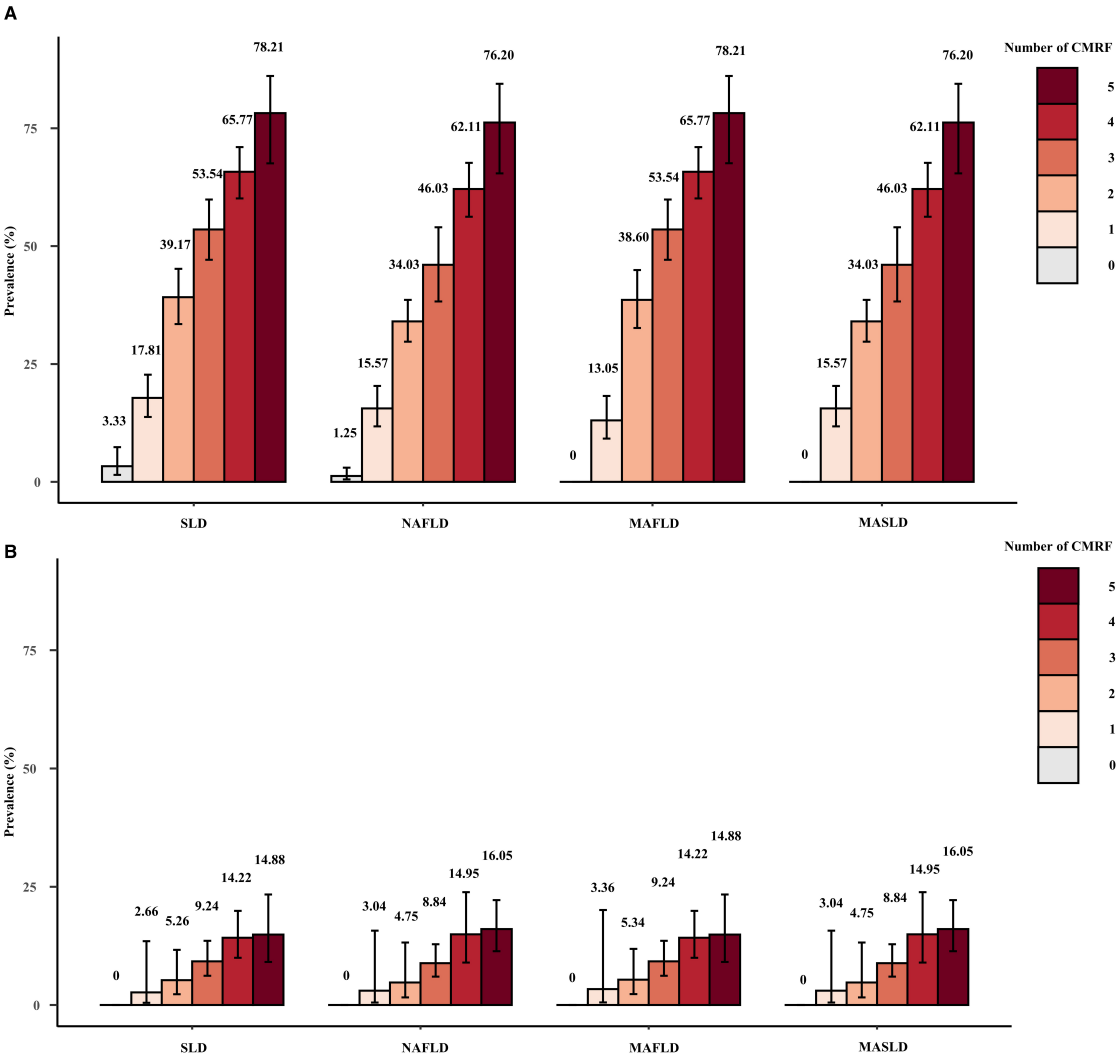
Weighted prevalence of SLD (CAP ≥274 dB/m), NAFLD, MAFLD, and MASLD **(A)** and associated prevalence of ALF **(B)** among patients with different numbers of CMRFs.

Figure 4 displays the weighted prevalence of each CMRF as defined by the new criteria, along with the weighted prevalence of elevated HOMA-IR and elevated HRCRP as defined by the previous diagnostic criteria for MAFLD. In NAFLD, MAFLD, and MASLD, elevated BMI/WC emerged as the prevailing metabolic disorder factor, whereas reduced HDL/drug treatment was the least common. The results remained the same in the sensitivity analysis (Supplementary Figure 4). Interestingly, we found that NAFLD/MAFLD/MASLD and the corresponding ALF prevalence increased as the number of CMRFs increased (Figure 5). Notably, the results remained consistent across the sensitivity analyses employing varying CAP values (Supplementary Figures 5, 6).

### Liver fibrosis under different definitions of fatty liver disease

Regardless of the disease definition, the LSM, FAST score, prevalence of fibrosis at all levels, and prevalence of progressive NASH (FAST ≥0.67) were significantly greater in the NAFLD/MAFLD/MASLD group than in the control group. However, the prevalence of liver fibrosis or progressive NASH was very similar across NAFLD, MAFLD, and MASLD, including within distinct racial subgroups (Table 1; Figures 2, 3).

Weighted logistic regression analyses were employed to evaluate the risk factors linked to ALF/progressive NASH, and the corresponding outcomes are presented in Table 2. Our findings indicate that after adjustment for confounding variables, the risk of ALF or progressive NASH was significantly greater in the NAFLD/MAFLD/MASLD group than in the control group. Furthermore, as depicted in Supplementary Tables 3, 5, this significant positive correlation remained in the sensitivity analysis for different CAPs.

**Table 2:** Weighted logistic regression analyses of the relationship between NAFLD/MAFLD/NAFLD and ALF/progressive NASH.

	**ALF**		**Progressive NASH**	
	**Minimally adjusted model**	**Fully adjusted model**	**Minimally adjusted model**	**Fully adjusted model**
	**(OR, 95% CI)**	**(OR, 95% CI)**	**(OR, 95% CI)**	**(OR, 95% CI)**
Non-NAFLD	Ref	Ref	Ref	Ref
NAFLD	3.76 (2.17, 6.53)	3.84 (2.28, 6.49)	3.81 (1.17, 12.41)	16.31 (5.93, 44.90)
Non-MAFLD	Ref	Ref	Ref	Ref
MAFLD	4.10 (2.27, 7.42)	3.61 (2.02, 6.44)	5.52 (1.09, 27.97)	16.38 (4.08, 65.72)
Non-MASLD	Ref	Ref	Ref	Ref
MASLD	3.78 (2.18, 6.56)	3.86 (2.28, 6.51)	3.83 (1.17, 12.53)	16.33 (5.94, 44.90)

Minimally adjusted model adjusted for age, sex, race, and smoking status. Fully adjusted model adjusted for age, sex, race, smoking status, ALT, ALP, ALB, GGT, UA, TC, and PLT.

ALF defined as LSM ≥ 9.7 kPa, progressive NASH defined as FAST ≥ 0.67.

## Discussion

According to our national survey, the weighted prevalence of SLD was estimated to be 41.21% (95% CI 40.52–44.34%), with the majority of SLD categorized as MASLD [37.90% (35.70–40.14%)]. Furthermore, MASLD consistently constituted the largest proportion of patients with SLD across the various CAP thresholds. Similar results were obtained in two articles that also applied NHANES data (8, 9). Therefore, this study focused on systematically comparing the similarities and differences among three nomenclatures: NAFLD, MAFLD, and MASLD. We found the following interesting conclusions, which still hold true at different CAP thresholds: First, the prevalence of NAFLD, MAFLD, and MASLD was similar, with MAFLD having the highest prevalence, which remained true across races, and excessive alcohol consumption or viral hepatitis being present in patients who met the diagnostic criteria for MAFLD, but not NAFLD or MASLD. Second, the clinical characteristics of all three groups were also relatively similar; patients with NAFLD/MAFLD/MASLD were significantly older, had a greater proportion of males, and had significantly greater WC, BMI, liver enzymes, blood glucose, and lipids than patients with non-steatotic liver disease. Third, the most common metabolic dysfunction factor, whether NAFLD, MAFLD, or MASLD, was elevated BMI/WC, whereas the least common factor was reduced HDL/drug treatment. Fourth, as the number of CMRFs increased, the prevalence of NAFLD/MAFLD/MASLD and the corresponding prevalence of ALF subsequently increased. Fifth, the prevalence of ALF and progressive NASH was similar in NAFLD, MAFLD, and MASLD, and the risk of ALF and progressive NASH was significantly greater in NAFLD, MAFLD, and MASLD than in non-fatty liver disease patients.

In this study, 2.04 and 1.74% of the weighted percentages of patients were consistent with a diagnosis of NAFLD and MASLD, respectively, but not with a diagnosis of MAFLD. Patients who were excluded from the MAFLD diagnosis because they were not overweight or obese, had diabetes, or had two or more metabolic disorders were excluded from the MAFLD diagnosis. Abdominal obesity was defined as a WC exceeding 94 cm for men and 88 cm for women according to the most recent consensus in nomenclature. However, the consensus does not offer specific guidelines for race-based adjustments. Consequently, this study relied solely on the aforementioned criteria for defining abdominal obesity. According to the diagnostic criteria for MAFLD, abdominal obesity was defined as a WC surpassing 102/88 cm in Caucasians and 90/80 cm in Asians. The disparity in the delineation of abdominal obesity between the two diagnostic criteria led to 46.15% of the non-MAFLD MASLD patients in this study satisfying the MASLD criteria for abdominal obesity while failing to meet the MAFLD criteria for abdominal obesity. Furthermore, 90.83% of the total population met the definition of CMRF according to the MASLD criteria, whereas only 82.71% met the diagnosis of overweight/obesity, diabetes, or two or more metabolic disorders according to MAFLD criteria. MAFLD patients who do not meet the diagnostic criteria for NAFLD or MASLD can be classified as MetALD or MASLD-viral, according to the new nomenclature consensus. The new consensus definition of metabolic disorders will include more patients with metabolic abnormalities than the original MAFLD diagnosis, although the changes brought about will be very small. In subsequent updates to the consensus, the definitions of each CMRF should be more detailed, such as how the definitions of overweight/obesity and abdominal obesity should be adjusted for different races to better guide clinical application. In general, a substantial level of agreement was observed among NAFLD, MAFLD, and MASLD, a finding that has been corroborated in different articles (8, 9, 23–25). Hence, it is our contention that while there remains ongoing debate regarding the utilization of terms MAFLD and MASLD, the renaming of NAFLD is the general trend. The coexistence of the terms NAFLD, MAFLD, and MASLD within academic discourse is anticipated until the implementation of new ICD codes by the World Health Organization and the rebranding of NAFLD and NASH by regulatory agencies in the United States and European Union. Based on our findings, the terminology of MASLD will facilitate the development of the field while maintaining the validity of the results of studies conducted over the past decades using the term NAFLD.

According to the present study, the NAFLD/MAFLD/MASLD and ALF prevalence increased with the number of CMRFs. Among the subjects eligible for all five CMRFs, the prevalence of NAFLD, MAFLD, and MASLD was >76%, and the prevalence of ALF was >14%. In a study by Yang et al., the prevalence of MASLD was as high as 83.7% and the prevalence of ALF was 24.3% in those who also fulfilled the five CMRFs, which stems from the fact that different CAP values and LSMs were used to diagnose significant hepatic steatosis and ALF (26). Liver fibrosis, particularly ALF, has been extensively documented to be significantly correlated with an unfavorable prognosis and is widely regarded as a reliable prognostic indicator for SLD (27–29). The pathogenesis and progression of NAFLD/MAFLD/MASLD are closely related to metabolic factors, and the inclusion of metabolic abnormalities in the diagnosis of MAFLD and MASLD is the recognition of metabolic abnormalities as a core aspect of disease development. Combined with the results of this study, we believe that in MASLD screening, we should focus on patients who meet a higher number of CMRFs and who have a greater likelihood of having MASLD as well as ALF, and that this approach is more conducive to the rational allocation of healthcare resources.

Importantly, our study has several limitations. First, due to the impracticality and invasive nature of liver biopsy in such a large number of samples, we could not use this diagnostic gold standard in our study. Second, the NHANES database lacks information on other etiologies that may contribute to hepatic steatosis, such as lysosomal acid lipase deficiency, Wilson's disease, hypobetalipoproteinemia, and inborn errors of metabolism. Third, weekly alcohol consumption was calculated based on self-reported alcohol consumption over the past year, which may have been subject to recall bias. Fourth, in observational studies, it is important to acknowledge that confounding factors may have not been measured or assessed, thus introducing the potential for residual confounding.

It is important to acknowledge the limitations of this study. Firstly, the utilization of CAP and LSM values from VCTE for diagnosing hepatic steatosis and severity of hepatic fibrosis may result in underdiagnosis and misdiagnosis when compared to the gold standard liver biopsy. However, the invasive nature of liver biopsy makes it challenging to apply to a population of this magnitude. Secondly, as NHANES is a general health survey, it does not provide a precise definition of rare causes of liver disease. Thirdly, while this study made efforts to control for confounding factors by adjusting for numerous potential confounders in the logistic regression modeling analysis, it did not encompass all pertinent variables that could potentially influence MASLD/ALF, including dietary structure, exercise, sleep, and other conditions. As a result, the accuracy of the findings may have been affected.

## Conclusion

In conclusion, our study showed a high degree of consistency among NAFLD, MAFLD, and MASLD. We believe that the new MASLD definition will not overrule the results of previous NAFLD-related studies, and that this new nomenclature, without stigmatization or certainty of diagnosis, will facilitate the identification and management of this disease. In addition, more attention should be given to patients who have a greater number of CMRFs and are at a greater risk of ALF.

## Data availability statement

The raw data supporting the conclusions of this article will be made available by the authors, without undue reservation.

## Ethics statement

The studies involving humans were approved by all participants were required to complete consent forms before engaging in the study, and the survey protocol received approval from the Ethics Review Board of the National Center for Health Statistics (NCHS) Research. The studies were conducted in accordance with the local legislation and institutional requirements. The participants provided their written informed consent to participate in this study.

## Author contributions

HZ: Data curation, Funding acquisition, Methodology, Software, Writing – original draft, Writing – review & editing. XM: Methodology, Supervision, Writing – review & editing. WP: Supervision, Writing – review & editing. YX: Funding acquisition, Investigation, Supervision, Writing – review & editing.
