# Supplementary material for: Comparing similarities and differences between NAFLD, MAFLD, and MASLD in the general U.S. population
Source: Front Nutr. 2024 Jul 8;11:1411802. doi: 10.3389/fnut.2024.1411802 (PMC11260733; doi:10.3389/fnut.2024.1411802)
Supplement: Supplementary file 1 [file Data_Sheet_1.docx]

Comparing similarities and differences between NAFLD, MAFLD, and MASLD in the general U.S. population

Supplementary material

1. Laboratory measurement and clinical data…………………………………………………………………….….1
2. Formula for calculating the FAST score…………………………………………………………………………..2
3. The definition of SLD………………………………………………………………………………………….….2
4. The definition of MASLD………………………………………………………………………………….….…..3
5. The definition of MetALD………………………………………………………………………………….….….3
6. The definition of ALD……………………………………………………………………………………….…….3
7. The definition of MASLD-viral………………………………………………………………………………..….3
8. The definition of cryptogenic SLD……………………………………………………………………………..…3
9. The definition of NAFLD………………………………………………………………………………………....3
10. The definition of MAFLD………………………………………………………………………………………...4
11. Supplement tables………………………………………………………………..………………………………..5
12. Supplement figures…………………………………………………………………………………………..…...12
13. Referrences…………………………………………………………………………………………………….…18

**Laboratory measurement and clinical data**

The NHANES dataset served as the source for all variables. These variables included demographic parameters (age, sex, and race), anthropometric parameters (waist circumference (WC) and body mass index (BMI)), lifestyles (smoking and alcohol consumption), vibration-controlled transient elastography (VCTE) parameters (liver stiffness measurements (LSM) and controlled attenuation parameter (CAP)), comorbidities (hypertension, diabetes, and viral hepatitis), and biomarkers such as platelets (PLT), total bilirubin (TBIL), alanine aminotransferase (ALT), aspartate aminotransferase (AST), γ-glutamyl transpeptidase (GGT), alkaline phosphatase (ALP), albumin (ALB), hemoglobin A1c (HbA1c), fasting plasma glucose (FPG), total cholesterol (TC), triglyceride (TG), high-density lipoprotein cholesterol (HDL), fasting insulin (FINS), uric acid (UA), creatinine (CRE), hypersensitive C reactive protein (HSCRP).

We categorized race into five groups: Hispanic, non-Hispanic Asian, non-Hispanic Black, non-Hispanic White, and other races. Besides, we categorized smoking into three groups (never: smoked <100 cigarettes in a lifetime, former: smoked ≥100 cigarettes in a lifetime and smoke not at all now, and current: smoked ≥100 cigarettes in a lifetime and smoke some days or every day) [1]. The calculation of the average weekly alcohol consumption was determined by considering the frequency of drinking days within the previous year and the mean quantity of alcohol consumed on those specific days (ALQ121 and ALQ130) [2]. Alcohol intake in the NHANES Alcohol Questionnaire is measured in standard drinks, which we converted to grams based on the conversion standard of 1 standard drink = 14 grams [3]. Excessive alcohol intake was defined as drinking more than 210g of alcohol per week for men and 140g for women [4]. The diagnostic criteria for diabetes were glycohemoglobin (HbA1c) > 6.5% or random plasma glucose ≥ 11.1 mmol/L or FPG ≥ 7.0 mmol/L or two-hour oral glucose tolerance test (OGTT) plasma glucose ≥ 11.1 mmol/L or under anti-diabetes therapy, or self-reported diabetes [5]. The definition of hypertension was based on systolic blood pressure (SBP) greater than 140 mmHg or diastolic blood pressure (DBP) greater than 90 mmHg, self-reported hypertension, or under antihypertension treatment [6]. Viral hepatitis was defined as the presence of hepatitis C virus infection, indicated by a positive test for viral RNA and/or antibodies, as well as hepatitis B virus infection, indicated by a positive test for surface antigen [7].

**Formula for calculating the FAST score**

FAST = (exp (-1.65 + 1.07 × log(LSM (Kpa)) + 2.66 × 10^-8^ × (CAP (dB/m))^3^ - 63.3 × (AST (U/L))^-1^)) / (1 + exp (-1.65 + 1.07 × log(LSM (Kpa)) + 2.66 × 10^-8^ × (CAP (dB/m))^3^ - 63.3 × (AST (U/L))^-1^))

**The definition of SLD**

The presence of significant hepatic steatosis (CAP ≥ 274 dB/m, or ≥ 248/302 dB/m for sensitivity analysis) was determined as SLD based on the most recent Delphi consensus [4].

**The definition of MASLD**

MASLD was defined as the presence of significant hepatic steatosis with one or more of the cardiometabolic risk factors (CMRF):

1) BMI ≥ 25 kg/m^2^ (≥ 23 kg/m^2^ for Asia) OR WC > 94 cm (for male), 80 cm (for female) OR ethnicity adjusted;

2) FPG ≥ 5.6 mmol/L OR two-hour OGTT plasma glucose ≥ 7.8 mmol/L OR HbA1c ≥ 5.7% OR type 2 diabetes OR treatment for type 2 diabetes;

3) Blood pressure ≥ 130/85 mmHg OR specific antihypertensive drug treatment;

4) TG ≥ 1.70 mmol/L OR lipid lowering treatment;

5) HDL ≤ 1.0 mmol/L for men OR ≤ 1.3 mmol/L for women OR lipid lowering treatment.

Patients with excessive alcohol consumption (> 140g per week for women, > 210g per week for men) and other causes of hepatic steatosis were excluded.

**The definition of MetALD**

MetALD was defined as the presence of excessive alcohol consumption on the basis of MASLD.

**The definition of ALD**

ALD was defined as the presence of more than moderate alcohol consumption on the basis of SLD, but without the presence of CMRF.

**The definition of MASLD-viral**

MASLD-other etiology was defined as the presence of viral hepatitis on the basis of MASLD.

**The definition of cryptogenic SLD**

Cryptogenic SLD refers to the presence of SLD without CMRF, moderate/heavy alcohol consumption, and viral hepatitis.

**The definition of NAFLD**

NAFLD was defined by the presence of significant hepatic steatosis and the absence of excessive alcohol consumption and other causes of hepatic steatosis.

**The definition of MAFLD**

MAFLD was defined as the presence of hepatic steatosis with one or more of the following [8-10]:

1) Overweight or obesity (BMI ≥25 kg/m^2^ (≥23 kg/m^2^ for Asia));

2) Diabetes;

3) All of the following items at least meet two metabolic abnormalities:

a. WC ≥102 cm in men and ≥ 88 cm in women;

b. Blood pressure ≥ 130/85 mmHg;

c. TG ≥ 1.70 mmol/L;

d. HDL < 1.0 mmol/L for men and <1.3 mmol/L for women;

e. Prediabetes (i.e., FPG 5.6 to 6.9 mmol/L, or HbA1c 5.7% to 6.4%);

f. HOMA-IR score ≥ 2.5;

g. HSCRP ≥ 2 mg/L.

**Supplement Tables**

**Table S1** Demographic, clinical, and laboratory parameters of SLD subclassifications in NHANES 2017-2020.3.

|  | Non-SLD | MASLD | MetALD | ALD | MASLD-viral | Cryptogenic SLD |
| --- | --- | --- | --- | --- | --- | --- |
| N (weighted prevalence) | 1713 (57.58) | 1205 (37.90) | 92 (3.33) | 5 (0.19) | 14 (0.88) | 6 (0.11) |
| Age (years) | 44.27 (0.89) | 50.67 (0.84) | 52.23 (2.32) | 36.97 (8.78) | 56.94 (0.78) | 31.03 (9.29) |
| Male (%) | 47.25 (1.65) | 56.67 (2.48) | 53.16 (10.85) | 87.10 (12.90) | 52.37 (6.77) | 68.88 (19.75) |
| WC (cm) | 92.14 (0.56) | 111.65 (0.80) | 104.16 (1.70) | 81.61 (3.64) | 107.69 (0.94) | 75.93 (3.14) |
| BMI (kg/m^2^) | 26.54 (0.24) | 34.08 (0.31) | 29.79 (0.77) | 22.05 (0.58) | 30.57 (0.53) | 21.96 (0.67) |
| PLT (10^9^/L) | 240.52 (2.28) | 242.75 (3.00) | 243.42 (7.90) | 294.63 (18.09) | 277.28 (10.81) | 214.80 (15.03) |
| TBIL (mg/dL) | 0.53 (0.02) | 0.49 (0.01) | 0.44 (0.03) | 0.32 (0.06) | 0.55 (0.03) | 0.51 (0.08) |
| ALT (U/L) | 19.77 (0.51) | 26.00 (0.76) | 31.49 (2.06) | 19.05 (4.36) | 30.48 (2.43) | 13.05 (3.13) |
| AST (U/L) | 20.86 (0.40) | 21.85 (0.48) | 30.57 (2.55) | 26.57 (5.29) | 33.09 (1.48) | 17.17 (1.13) |
| GGT (U/L) | 24.10 (0.72) | 32.99 (1.24) | 51.43 (6.60) | 21.21 (3.37) | 34.14 (4.54) | 13.32 (1.19) |
| ALP (U/L) | 71.82 (0.89) | 77.51 (0.97) | 76.47 (4.97) | 95.98 (6.62) | 95.43 (2.82) | 86.82 (5.27) |
| ALB (g/dL) | 41.30 (0.17) | 40.09 (0.19) | 40.41 (0.49) | 42.29 (2.02) | 41.25 (0.48) | 40.43 (1.65) |
| HbA1C (%) | 5.45 (0.02) | 5.96 (0.05) | 5.55 (0.08) | 5.30 (0.11) | 5.72 (0.13) | 5.30 (0.04) |
| FPG (mg/dL) | 94.86 (0.54) | 111.74(1.77) | 99.50 (2.22) | 86.84 (2.42) | 110.11(3.06) | 88.45 (2.20) |
| TG (mg/dL) | 101.01 (1.42) | 154.68 (4.84) | 171.14 (16.84) | 100.45 (11.26) | 118.16 (5.54) | 84.13 (12.03) |
| TC (mg/dL) | 182.46 (1.54) | 186.47 (2.65) | 208.51 (5.52) | 190.81 (12.10) | 193.39 (4.15) | 156.37 (8.62) |
| HDL (mg/dL) | 57.39 (0.58) | 48.16 (0.73) | 60.10 (2.71) | 67.86 (6.16) | 53.06 (1.64) | 52.56 (3.16) |
| FINS | 9.31 (0.31) | 19.91 (1.01) | 11.86 (1.70) | 5.23 (0.34) | 21.19 (0.81) | 5.67 (0.82) |
| HOMA-IR | 2.50 (0.12) | 6.17 (0.40) | 3.32 (0.53) | 1.23 (0.09) | 6.16 (0.32) | 1.30 (0.15) |
| CRE (mg/dL) | 0.86 (0.01) | 0.87 (0.01) | 0.85 (0.03) | 0.88 (0.04) | 0.80 (0.04) | 0.98 (0.10) |
| UA (mg/dL) | 5.11 (0.05) | 5.84 (0.04) | 6.07 (0.27) | 4.96 (0.82) | 6.20 (0.14) | 4.83 (0.47) |
| HSCRP (mg/L) | 3.09 (0.28) | 4.81 (0.25) | 4.01 (0.73) | 2.14 (0.91) | 4.73 (2.78) | 1.27 (0.76) |
| CAP (dB/m) | 220.56 (1.24) | 323.89 (1.84) | 316.48 (4.08) | 280.34 (2.22) | 323.31 (3.79) | 297.28 (5.53) |
| LSM (kPa) | 5.09 (0.08) | 6.67 (0.28) | 6.06 (0.35) | 5.83 (0.87) | 7.08 (2.01) | 5.39 (0.28) |
| FAST | 0.07 (0.00) | 0.17 (0.01) | 0.23 (0.03) | 0.15 (0.05) | 0.31 (0.03) | 0.06 (0.01) |
| Smoking |  |  |  |  |  |  |
| Never | 56.37 (2.06) | 55.55 (2.25) | 24.27 (6.93) | 46.28(25.44) | 3.64 (4.46) | 61.46(20.92) |
| Former | 25.85 (1.60) | 31.78 (2.20) | 41.51(11.04) | 27.83(23.30) | 4.65 (4.44) | 38.54(20.92) |
| Current | 17.78 (1.81) | 12.67 (1.74) | 34.22 (6.90) | 25.89(20.61) | 91.72 (7.56) | 0.00 (0.00) |
| Excessive alcohol intake | 7.73 (1.26) | 0.00 (0.00) | 100.00(0.00) | 100.00(0.00) | 0.00 (0.00) | 0.00 (0.00) |
| Hypertension | 23.25 (1.55) | 43.47 (2.73) | 31.39 (4.52) | 0.00 (0.00) | 14.38(12.10) | 0.00 (0.00) |
| DM | 7.98 (0.82) | 27.01 (1.72) | 14.26 (2.79) | 0.00 (0.00) | 12.03(10.44) | 0.00 (0.00) |

Note: Continuous variables are shown as mean (standard error of mean (SE)). Categorical values are shown as % (SE). Abbreviations: WC, waist circumference; BMI, body mass index; PLT, platelets; TBIL, total bilirubin; ALT, alanine aminotransferase; AST, aspartate aminotransferase; GGT, γ-glutamyl transpeptidase; ALP, alkaline phosphatase; ALB, albumin; HbA1c, hemoglobin A1c; FPG, fasting plasma glucose; TC, total cholesterol; TG, triglyceride; HDL, high-density lipoprotein cholesterol; FINS, fasting insulin; HOMA-IR, homeostasis model assessment of insulin resistance; UA, uric acid; CRE, creatinine; HSCRP, hypersensitive C reactive protein; CAP, controlled attenuation parameter; LSM, liver stiffness measurements; FAST, FibroScan-AST.

**Table S2** Demographic, clinical, and laboratory parameters of NAFLD, MAFLD, and MASLD (CAP ≥248 dB/m) in NHANES 2017-2020.3.

| variable | Non-NAFLD | NAFLD | Non-MAFLD | MAFLD | Non-MASLD | MASLD |
| --- | --- | --- | --- | --- | --- | --- |
| N (weighted prevalence) | 1414 (48.41) | 1621 (51.59) | 1331 (45.65) | 1704 (54.35) | 1435 (49.41) | 1600 (50.59) |
| Age (years) | 43.38 (0.87) | 50.48 (0.86) ^a^ | 42.31 (0.98) | 51.01 (0.76) ^a^ | 43.13 (0.87) | 50.86 (0.80) ^a^ |
| Male (%) | 47.21 (1.96) | 54.87 (2.20) ^a^ | 46.39 (2.19) | 55.18 (1.87) ^a^ | 47.96 (1.94) | 54.29 (2.06) ^a^ |
| Race (%) |  |  |  |  |  |  |
| Non-Hispanic Black | 12.67 (1.50) | 8.84 (1.36) ^a^ | 12.63 (1.57) | 9.07 (1.34) ^a^ | 12.51 (1.49) | 8.93 (1.38) ^a^ |
| Non-Hispanic White | 64.37 (2.14) | 64.02 (2.39) | 64.16 (2.26) | 64.21 (2.19) | 64.57 (2.12) | 63.82 (2.47) |
| Hispanic | 13.96 (1.63) | 18.13 (1.76) ^a^ | 14.17 (1.51) | 17.74 (1.87) ^a^ | 13.93 (1.56) | 18.24 (1.89) ^a^ |
| Non-Hispanic Asian | 4.74 (0.84) | 4.08 (0.79) | 4.96 (0.88) | 3.93 (0.77) ^a^ | 4.74 (0.82) | 4.06 (0.79) |
| Other | 4.26 (0.64) | 4.93 (0.87) | 4.07 (0.56) | 5.05 (0.88) | 4.26 (0.64) | 4.95 (0.89) |
| WC (cm) | 91.13 (0.53) | 108.39 (0.71) ^a^ | 89.07 (0.56) | 109.24 (0.61) ^a^ | 90.89 (0.56) | 108.97 (0.66) ^a^ |
| BMI (kg/m^2^) | 26.10 (0.22) | 32.74 (0.30) ^a^ | 25.47 (0.23) | 32.93 (0.26) ^a^ | 26.00 (0.24) | 32.96 (0.27) ^a^ |
| PLT (10^9^/L) | 241.19 (2.55) | 242.49 (2.53) | 238.81 (2.64) | 244.42 (2.34) | 240.80 (2.54) | 242.89 (2.55) |
| TBIL (mg/dL) | 0.52 (0.02) | 0.50 (0.01) | 0.53 (0.02) | 0.49 (0.01) ^a^ | 0.52 (0.02) | 0.50 (0.01) |
| ALT (U/L) | 20.51 (0.74) | 24.57 (0.58) ^a^ | 18.82 (0.60) | 25.78 (0.63) ^a^ | 20.40 (0.73) | 24.75 (0.59) ^a^ |
| AST (U/L) | 21.84 (0.63) | 21.52 (0.36) | 20.58 (0.39) | 22.59 (0.52) ^a^ | 21.75 (0.61) | 21.59 (0.37) |
| GGT (U/L) | 25.15 (0.90) | 31.55 (0.92) ^a^ | 21.64 (0.55) | 34.17 (0.98) ^a^ | 25.06 (0.90) | 31.76 (0.92) ^a^ |
| ALP (U/L) | 72.08 (1.13) | 76.57 (0.82) ^a^ | 70.83 (0.91) | 77.40 (0.74) ^a^ | 72.03 (1.09) | 76.72 (0.80) ^a^ |
| ALB (g/dL) | 41.34 (0.19) | 40.32 (0.16) ^a^ | 41.52 (0.18) | 40.22 (0.16) ^a^ | 41.41 (0.18) | 40.23 (0.15) ^a^ |
| HbA1C (%) | 5.41 (0.02) | 5.86 (0.05) ^a^ | 5.38 (0.02) | 5.87 (0.05) ^a^ | 5.41 (0.02) | 5.88 (0.05) ^a^ |
| FPG (mg/dL) | 93.65 (0.68) | 108.90 (1.41) ^a^ | 92.50 (0.57) | 109.10 (1.35) ^a^ | 93.54 (0.69) | 109.32 (1.42) ^a^ |
| TG (mg/dL) | 100.22 (2.10) | 145.95 (3.84) ^a^ | 92.91 (1.40) | 149.77 (4.04) ^a^ | 99.51 (2.12) | 147.55 (3.95) ^a^ |
| TC (mg/dL) | 183.14 (1.99) | 186.61 (2.17) | 181.31 (2.06) | 187.97 (2.24) ^a^ | 182.67 (1.91) | 187.13 (2.25) |
| HDL (mg/dL) | 58.75 (0.70) | 49.47 (0.60) ^a^ | 59.43 (0.76) | 49.37 (0.70) ^a^ | 58.82 (0.67) | 49.21 (0.63) ^a^ |
| FINS (μU/mL) | 8.74 (0.36) | 17.98 (0.88) ^a^ | 7.94 (0.27) | 18.18 (0.74) ^a^ | 8.65 (0.36) | 18.25 (0.81) ^a^ |
| HOMA-IR | 2.27 (0.11) | 5.52 (0.35) ^a^ | 2.01 (0.08) | 5.57 (0.30) ^a^ | 2.25 (0.11) | 5.61 (0.33) ^a^ |
| CRE (mg/dL) | 0.86 (0.01) | 0.87 (0.01) | 0.86 (0.01) | 0.87 (0.01) | 0.86 (0.01) | 0.87 (0.01) ^a^ |
| UA (mg/dL) | 5.13 (0.07) | 5.72 (0.04) ^a^ | 5.00 (0.06) | 5.79 (0.04) ^a^ | 5.12 (0.07) | 5.73 (0.04) ^a^ |
| HSCRP (mg/L) | 3.00 (0.24) | 4.52 (0.30) ^a^ | 2.67 (0.26) | 4.72 (0.28) ^a^ | 2.95 (0.23) | 4.60 (0.30) ^a^ |
| CAP (dB/m) | 218.01 (1.97) | 307.19 (1.75) ^a^ | 210.74 (1.60) | 308.76 (1.59) ^a^ | 218.88 (1.82) | 308.11 (1.61) ^a^ |
| LSM (kPa) | 5.15 (0.10) | 6.29 (0.21) ^a^ | 5.03 (0.08) | 6.34 (0.20) ^a^ | 5.19 (0.09) | 6.28 (0.21) ^a^ |
| FAST | 0.08 (0.01) | 0.14 (0.01) ^a^ | 0.06 (0.00) | 0.16 (0.01) ^a^ | 0.08 (0.01) | 0.14 (0.01) ^a^ |
| Smoking (%) |  |  |  |  |  |  |
| Never | 55.16 (1.91) | 53.90 (1.62) | 59.13 (2.28) | 50.63 (1.89) ^a^ | 54.98 (1.82) | 54.06 (1.58) |
| Former | 24.01 (1.98) | 32.62 (1.45) ^a^ | 23.76 (1.92) | 32.40 (1.16) ^a^ | 24.28 (1.90) | 32.53 (1.29) ^a^ |
| Current | 20.83 (1.50) | 13.48 (1.26) ^a^ | 17.11 (1.72) | 16.97 (1.53) | 20.74 (1.45) | 13.42 (1.35) ^a^ |
| Viral hepatitis (%) | 4.62 (1.44) | 0.00 (0.00) ^a^ | 2.37 (0.58) | 2.13 (1.21) | 4.53 (1.41) | 0.00 (0.00) ^a^ |
| Excessive alcohol intake (%) | 16.47 (1.60) | 0.00 (0.00) ^a^ | 8.39 (1.45) | 7.63 (0.89) | 16.14 (1.66) | 0.00 (0.00) ^a^ |
| Hypertension (%) | 21.27 (1.90) | 40.19 (1.81) ^a^ | 19.03 (1.84) | 41.11 (2.16) ^a^ | 20.84 (1.79) | 40.99 (1.94) ^a^ |
| Diabetes (%) | 6.34 (0.73) | 23.92 (1.40) ^a^ | 5.07 (0.79) | 24.10 (1.40) ^a^ | 6.21 (0.73) | 24.40 (1.32) ^a^ |
| FAST ≥0.67 | 1.10 (0.57) | 2.65 (0.52) | 0.48 (0.29) | 3.09 (0.56) ^a^ | 1.07 (0.56) | 2.71 (0.53) |
| ≥F2 (%) | 4.49 (0.69) | 12.04 (1.48) ^a^ | 3.40 (0.42) | 12.57 (1.49) ^a^ | 4.51 (0.66) | 12.16 (1.44) ^a^ |
| ≥F3 (%) | 2.63 (0.68) | 8.30 (1.36) ^a^ | 1.86 (0.50) | 8.67 (1.33) ^a^ | 2.70 (0.70) | 8.36 (1.35) ^a^ |
| F4 (%) | 1.38 (0.39) | 3.65 (0.74) ^a^ | 1.23 (0.46) | 3.66 (0.67) ^a^ | 1.47 (0.40) | 3.61 (0.72) ^a^ |

Note: Continuous variables are shown as mean (standard error of mean (SE)). Categorical values are shown as % (SE). Abbreviations: WC, waist circumference; BMI, body mass index; PLT, platelets; TBIL, total bilirubin; ALT, alanine aminotransferase; AST, aspartate aminotransferase; GGT, γ-glutamyl transpeptidase; ALP, alkaline phosphatase; ALB, albumin; HbA1c, hemoglobin A1c; FPG, fasting plasma glucose; TC, total cholesterol; TG, triglyceride; HDL, high-density lipoprotein cholesterol; FINS, fasting insulin; HOMA-IR, homeostasis model assessment of insulin resistance; UA, uric acid; CRE, creatinine; HSCRP, hypersensitive C reactive protein; CAP, controlled attenuation parameter; LSM, liver stiffness measurements; FAST, FibroScan-AST; F2, stage 2 fibrosis; F3, stage 3 fibrosis; F4, stage 4 fibrosis.

^a^ Significantly different from controls (*P* <0.05).

**Table S3** Weighted logistic regression analyses of the relationship between NAFLD/MAFLD/NAFLD (CAP ≥248 dB/m) and ALF/ progressive NASH.

|  | ALF | | progressive NASH | |
| --- | --- | --- | --- | --- |
|  | Minimally adjusted model  (OR, 95%CI) | Fully adjusted model  (OR, 95%CI) | Minimally adjusted model  (OR, 95%CI) | Fully adjusted model  (OR, 95%CI) |
| Non-NAFLD | Ref | Ref | Ref | Ref |
| NAFLD | 2.91 (1.60, 5.30) | 3.22 (1.98, 5.24) | 2.25 (0.62, 8.18) | 8.26 (2.49, 27.43) |
| Non-MAFLD | Ref | Ref | Ref | Ref |
| MAFLD | 4.52 (2.76, 7.41) | 3.72 (2.16, 6.42) | 6.48 (1.57, 26.71) | 14.48 (1.62, 129.45) |
| Non- MASLD | Ref | Ref | Ref | Ref |
| MASLD | 3.03 (1.67, 5.49) | 3.31 (2.05, 5.36) | 2.43 (0.66, 8.98) | 8.33 (2.53, 27.43) |

Note: Minimally adjusted model adjusted for age, sex, race, smoking status. Fully adjusted model adjusted for age, sex, race, smoking status, ALT, ALP, ALB, GGT, UA, TC, and PLT.

ALF defined as LSM ≥9.7 kPa, progressive NASH defined as FAST ≥0.67.

**Table S4** Demographic, clinical, and laboratory parameters of NAFLD, MAFLD, and MASLD (CAP ≥302 dB/m) in NHANES 2017-2020.3.

| variable | Non-NAFLD | NAFLD | Non-MAFLD | MAFLD | Non-MASLD | MASLD |
| --- | --- | --- | --- | --- | --- | --- |
| N (weighted prevalence) | 2243 (75.18) | 792 (24.82) | 2177 (73.13) | 858 (26.87) | 2245 (75.23) | 790 (24.77) |
| Age (years) | 45.88 (0.70) | 50.56 (0.95) ^a^ | 45.67 (0.73) | 50.79 (0.87) ^a^ | 45.88 (0.69) | 50.56 (0.95) ^a^ |
| Male (%) | 48.96 (1.58) | 57.83 (2.59) ^a^ | 48.25 (1.63) | 59.08 (2.08) ^a^ | 48.99 (1.60) | 57.76 (2.58) ^a^ |
| Race (%) |  |  |  |  |  |  |
| Non-Hispanic Black | 11.68 (1.43) | 7.72 (1.41) ^a^ | 11.72 (1.47) | 7.92 (1.39) ^a^ | 11.68 (1.43) | 7.73 (1.41) ^a^ |
| Non-Hispanic White | 63.97 (1.87) | 64.84 (3.26) | 63.92 (1.99) | 64.92 (2.97) | 63.96 (1.87) | 64.87 (3.28) |
| Hispanic | 15.13 (1.59) | 19.09 (2.20) ^a^ | 15.05 (1.59) | 19.00 (2.14) ^a^ | 15.15 (1.59) | 19.03 (2.23) ^a^ |
| Non-Hispanic Asian | 4.51 (0.79) | 4.08 (0.85) | 4.52 (0.79) | 4.08 (0.85) | 4.50 (0.79) | 4.09 (0.86) |
| Other | 4.71 (0.68) | 4.28 (1.29) | 4.80 (0.69) | 4.08 (1.20) | 4.71 (0.68) | 4.28 (1.29) |
| WC (cm) | 94.92 (0.48) | 115.54 (1.01) ^a^ | 94.45 (0.47) | 115.22 (1.01) ^a^ | 94.91 (0.47) | 115.60 (1.03) ^a^ |
| BMI (kg/m^2^) | 27.52 (0.21) | 35.61 (0.41) ^a^ | 27.38 (0.21) | 35.35 (0.41) ^a^ | 27.51 (0.21) | 35.64 (0.42) ^a^ |
| PLT (10^9^/L) | 241.87 (2.33) | 241.83 (3.34) | 242.07 (2.38) | 241.29 (3.04) | 241.84 (2.33) | 241.91 (3.34) |
| TBIL (mg/dL) | 0.52 (0.02) | 0.49 (0.01) | 0.52 (0.02) | 0.48 (0.01) | 0.52 (0.02) | 0.49 (0.01) |
| ALT (U/L) | 20.82 (0.49) | 28.02 (1.04) ^a^ | 20.28 (0.43) | 28.94 (1.07) ^a^ | 20.81 (0.49) | 28.06 (1.04) ^a^ |
| AST (U/L) | 21.32 (0.43) | 22.73 (0.59) ^a^ | 20.91 (0.35) | 23.76 (0.84) ^a^ | 21.32 (0.43) | 22.75 (0.59) ^a^ |
| GGT (U/L) | 25.98 (0.50) | 35.95 (1.66) ^a^ | 25.02 (0.58) | 37.79 (1.75) ^a^ | 25.97 (0.50) | 35.99 (1.67) ^a^ |
| ALP (U/L) | 72.94 (0.77) | 78.83 (1.22) ^a^ | 72.75 (0.75) | 78.89 (1.25) ^a^ | 72.95 (0.76) | 78.82 (1.22) ^a^ |
| ALB (g/dL) | 41.11 (0.16) | 39.91 (0.19) ^a^ | 41.14 (0.16) | 39.92 (0.18) ^a^ | 41.11 (0.16) | 39.91 (0.19) ^a^ |
| HbA1C (%) | 5.50 (0.03) | 6.08 (0.06) ^a^ | 5.49 (0.03) | 6.06 (0.05) ^a^ | 5.50 (0.03) | 6.08 (0.06) ^a^ |
| FPG (mg/dL) | 96.95 (0.64) | 115.37 (1.81) ^a^ | 96.55 (0.67) | 115.06 (1.71) ^a^ | 96.95 (0.64) | 115.42 (1.82) ^a^ |
| TG (mg/dL) | 110.36 (1.83) | 164.57 (6.10) ^a^ | 108.21 (1.72) | 166.28 (5.56) ^a^ | 110.35 (1.83) | 164.69 (6.09) ^a^ |
| TC (mg/dL) | 184.87 (1.56) | 185.10 (2.68) | 184.13 (1.50) | 187.10 (2.65) | 184.85 (1.57) | 185.16 (2.66) |
| HDL (mg/dL) | 56.57 (0.63) | 46.05 (0.78) ^a^ | 56.62 (0.65) | 46.72 (0.92) ^a^ | 56.57 (0.63) | 46.05 (0.78) ^a^ |
| FINS (μU/mL) | 10.47 (0.31) | 22.72 (1.41) ^a^ | 10.23 (0.30) | 22.43 (1.22) ^a^ | 10.46 (0.31) | 22.75 (1.41) ^a^ |
| HOMA-IR | 2.83 (0.10) | 7.33 (0.58) ^a^ | 2.75 (0.10) | 7.20 (0.49) ^a^ | 2.83 (0.10) | 7.34 (0.58) ^a^ |
| CRE (mg/dL) | 0.87 (0.01) | 0.87 (0.01) | 0.87 (0.01) | 0.87 (0.01) | 0.87 (0.01) | 0.87 (0.01) |
| UA (mg/dL) | 5.26 (0.05) | 5.94 (0.04) ^a^ | 5.23 (0.05) | 5.98 (0.05) ^a^ | 5.26 (0.05) | 5.94 (0.04) ^a^ |
| HSCRP (mg/L) | 3.19 (0.22) | 5.58 (0.34) ^a^ | 3.15 (0.22) | 5.50 (0.30) ^a^ | 3.19 (0.22) | 5.58 (0.34) ^a^ |
| CAP (dB/m) | 237.86 (1.19) | 343.26 (1.83) ^a^ | 234.79 (1.17) | 343.58 (1.82) ^a^ | 237.90 (1.19) | 343.32 (1.85) ^a^ |
| LSM (kPa) | 5.24 (0.08) | 7.25 (0.33) ^a^ | 5.17 (0.08) | 7.28 (0.32) ^a^ | 5.24 (0.08) | 7.25 (0.33) ^a^ |
| FAST | 0.08 (0.00) | 0.20 (0.01) ^a^ | 0.08 (0.00) | 0.22 (0.01) ^a^ | 0.08 (0.00) | 0.20 (0.01) ^a^ |
| Smoking (%) |  |  |  |  |  |  |
| Never | 55.26 (1.43) | 52.24 (2.53) | 56.42 (1.47) | 49.33 (2.44) ^a^ | 55.23 (1.44) | 52.33 (2.54) |
| Former | 26.18 (1.16) | 35.34 (2.31) ^a^ | 25.89 (1.24) | 35.42 (2.04) ^a^ | 26.22 (1.18) | 35.22 (2.32) ^a^ |
| Current | 18.56 (1.27) | 12.42 (1.88) ^a^ | 17.69 (1.34) | 15.25 (1.84) | 18.55 (1.27) | 12.45 (1.89) ^a^ |
| Viral hepatitis (%) | 2.98 (0.95) | 0.00 (0.00) | 2.43 (0.67) | 1.72 (1.11) | 2.98 (0.95) | 0.00 (0.00) |
| Excessive alcohol intake (%) | 10.61 (1.19) | 0.00 (0.00) ^a^ | 8.50 (1.11) | 6.53 (1.10) | 10.60 (1.19) | 0.00 (0.00) ^a^ |
| Hypertension (%) | 25.94 (1.57) | 46.46 (3.18) ^a^ | 25.86 (1.57) | 45.11 (3.29) ^a^ | 25.92 (1.57) | 46.55 (3.18) ^a^ |
| Diabetes (%) | 9.35 (0.75) | 33.77 (1.97) ^a^ | 9.01 (0.75) | 32.84 (1.97) ^a^ | 9.35 (0.75) | 33.83 (1.98) ^a^ |
| FAST ≥0.67 | 0.80 (0.36) | 5.24 (1.05) ^a^ | 0.68 (0.37) | 5.21 (0.98) ^a^ | 0.79 (0.36) | 5.25 (1.05) ^a^ |
| ≥F2 (%) | 4.75 (0.65) | 19.40 (2.10) ^a^ | 4.36 (0.62) | 19.34 (2.15) ^a^ | 4.74 (0.65) | 19.44 (2.11) ^a^ |
| ≥F3 (%) | 2.84 (0.64) | 13.80 (2.03) ^a^ | 2.63 (0.65) | 13.54 (1.99) ^a^ | 2.84 (0.64) | 13.82 (2.04) ^a^ |
| F4 (%) | 1.34 (0.28) | 6.23 (1.35) ^a^ | 1.17 (0.31) | 6.30 (1.28) ^a^ | 1.34 (0.28) | 6.24 (1.36) ^a^ |

Note: Continuous variables are shown as mean (standard error of mean (SE)). Categorical values are shown as % (SE). Abbreviations: WC, waist circumference; BMI, body mass index; PLT, platelets; TBIL, total bilirubin; ALT, alanine aminotransferase; AST, aspartate aminotransferase; GGT, γ-glutamyl transpeptidase; ALP, alkaline phosphatase; ALB, albumin; HbA1c, hemoglobin A1c; FPG, fasting plasma glucose; TC, total cholesterol; TG, triglyceride; HDL, high-density lipoprotein cholesterol; FINS, fasting insulin; HOMA-IR, homeostasis model assessment of insulin resistance; UA, uric acid; CRE, creatinine; HSCRP, hypersensitive C reactive protein; CAP, controlled attenuation parameter; LSM, liver stiffness measurements; FAST, FibroScan-AST; F2, stage 2 fibrosis; F3, stage 3 fibrosis; F4, stage 4 fibrosis.

^a^ Significantly different from controls (*P* <0.05).

**Table S5** Weighted logistic regression analyses of the relationship between NAFLD/MAFLD/NAFLD (CAP ≥302 dB/m) and ALF/ progressive NASH.

|  | ALF | | progressive NASH | |
| --- | --- | --- | --- | --- |
|  | Minimally adjusted model  (OR, 95%CI) | Fully adjusted model  (OR, 95%CI) | Minimally adjusted model  (OR, 95%CI) | Fully adjusted model  (OR, 95%CI) |
| Non-NAFLD | Ref | Ref | Ref | Ref |
| NAFLD | 5.09 (3.28, 7.92) | 4.95 (3.12, 7.85) | 6.47 (2.12, 19.72) | 23.50 (8.51, 64.90) |
| Non-MAFLD | Ref | Ref | Ref | Ref |
| MAFLD | 5.22 (3.22, 8.47) | 4.66 (2.87, 7.55) | 7.47 (1.93, 28.87) | 15.09 (5.32, 42.80) |
| Non- MASLD | Ref | Ref | Ref | Ref |
| MASLD | 5.11 (3.28, 7.95) | 4.96 (3.12, 7.88) | 6.50 (2.13, 19.83) | 23.52 (8.52, 64.92) |

Note: Minimally adjusted model adjusted for age, sex, race, smoking status. Fully adjusted model adjusted for age, sex, race, smoking status, ALT, ALP, ALB, GGT, UA, TC, and PLT.

ALF defined as LSM ≥9.7 kPa, progressive NASH defined as FAST ≥0.67.

**Supplement figures**

**Figure S1** (A) Weighted prevalence of SLD (CAP ≥ 248 dB/m), NAFLD, MAFLD, and MASLD and associated prevalence of ALF. (B) Weighted prevalence of SLD (CAP ≥ 248 dB/m), NAFLD, MAFLD, MASLD, and associated prevalence of ALF.

**
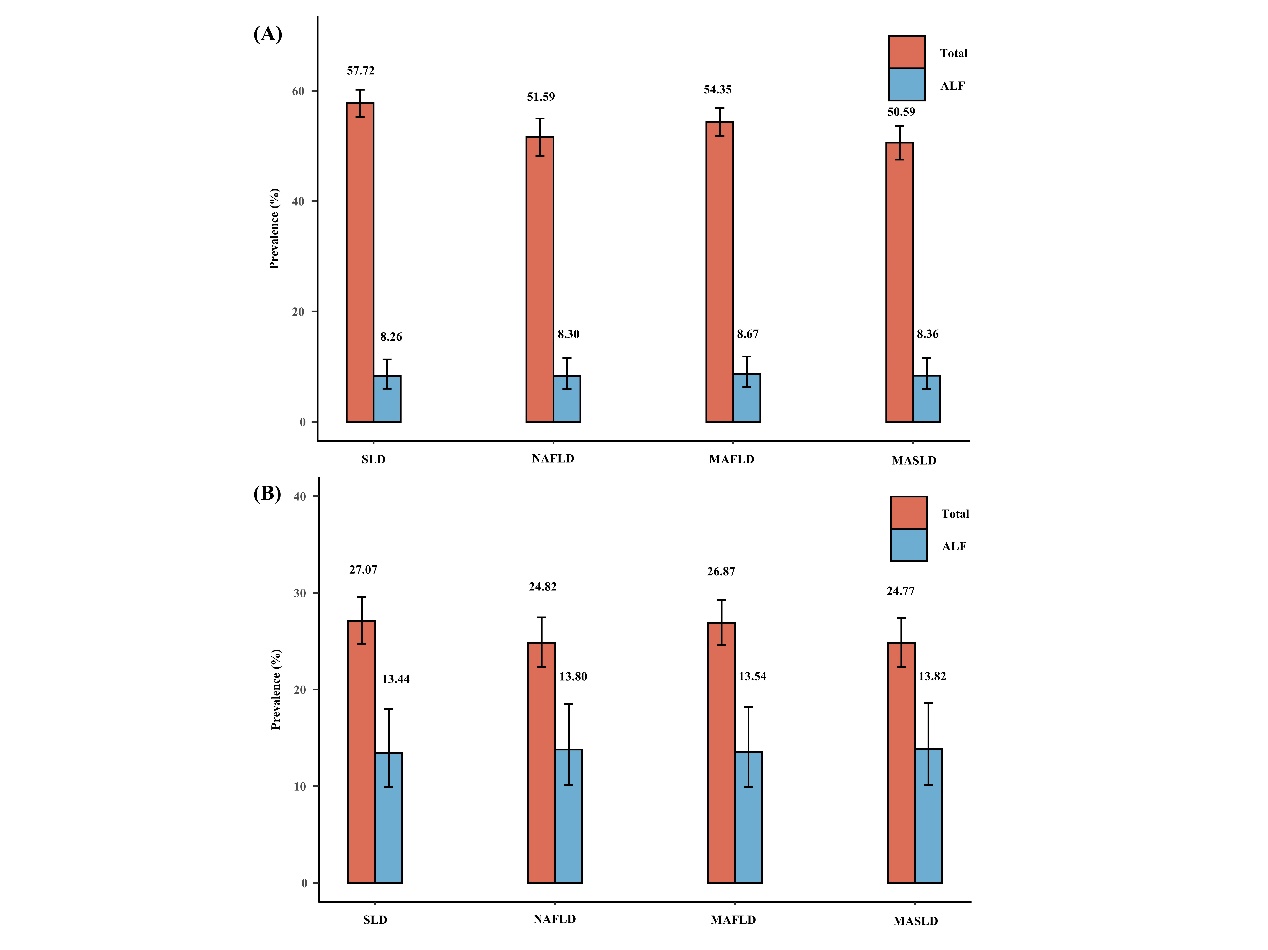
**

**Figure S2** Weighted prevalence of SLD (CAP ≥ 248 dB/m), NAFLD, MAFLD, and MASLD (A) and associated prevalence of ALF (B) in different races.


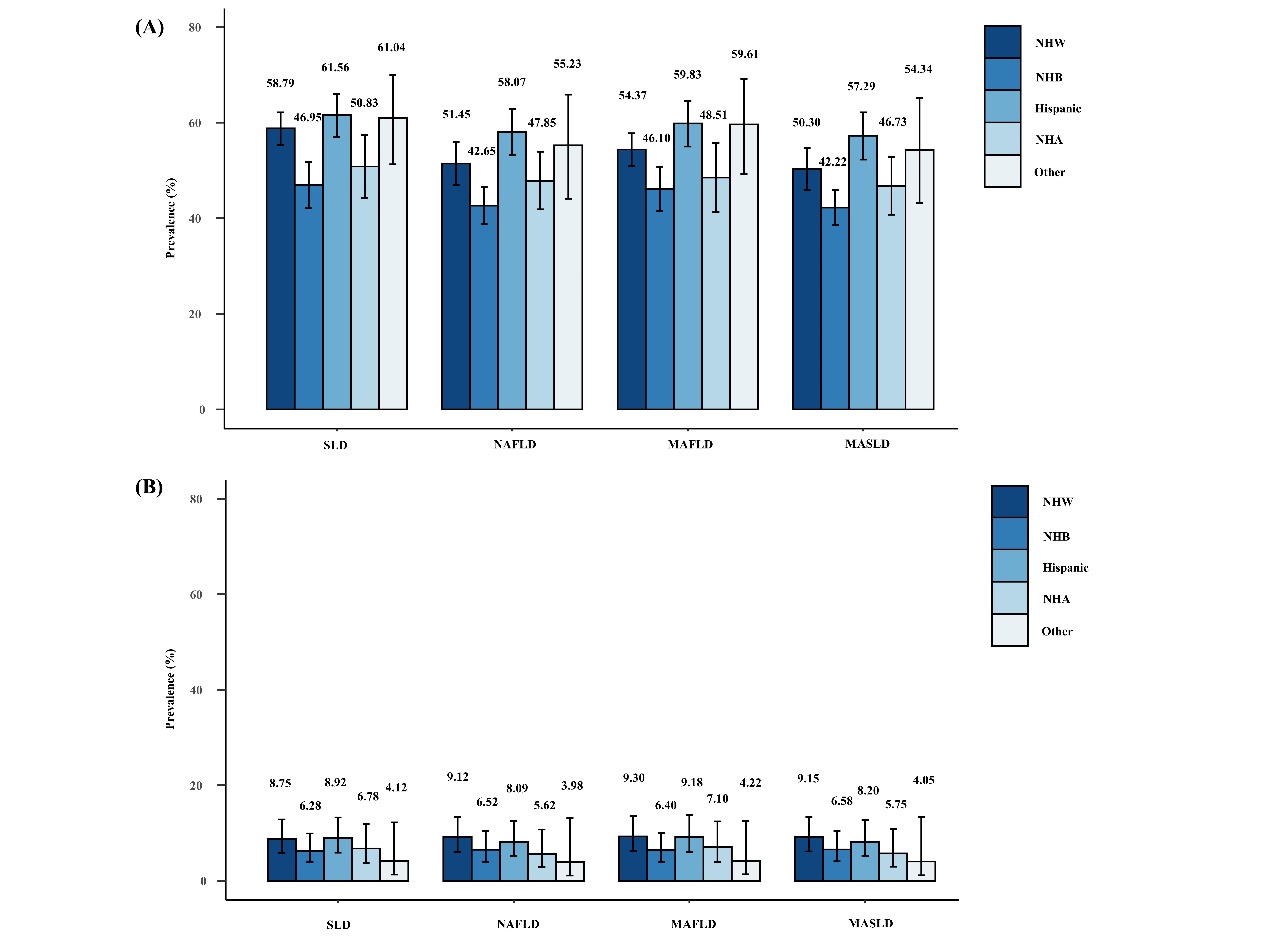


**Figure S3** Weighted prevalence of SLD (CAP ≥ 302 dB/m), NAFLD, MAFLD, and MASLD (A) and associated prevalence of ALF (B) in different races.

**
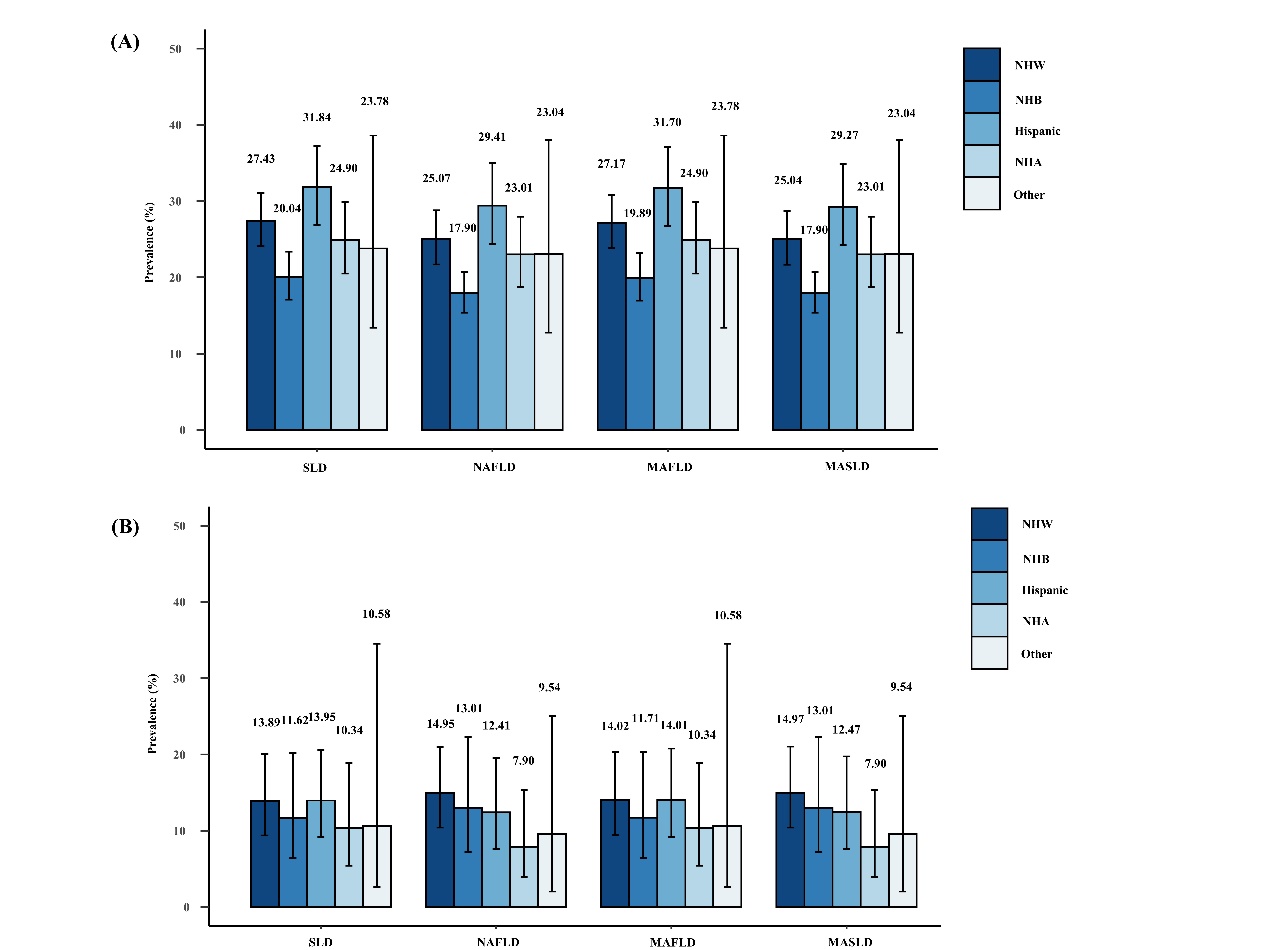
**

**Figure S4** (A) Weighted prevalence of each constituent of the CMRF definition among patients with SLD (CAP ≥ 248 dB/m), NAFLD, MAFLD, and MASLD. (B) Weighted prevalence of each constituent of the CMRF definition among patients with SLD (CAP ≥ 302 dB/m), NAFLD, MAFLD, and MASLD.


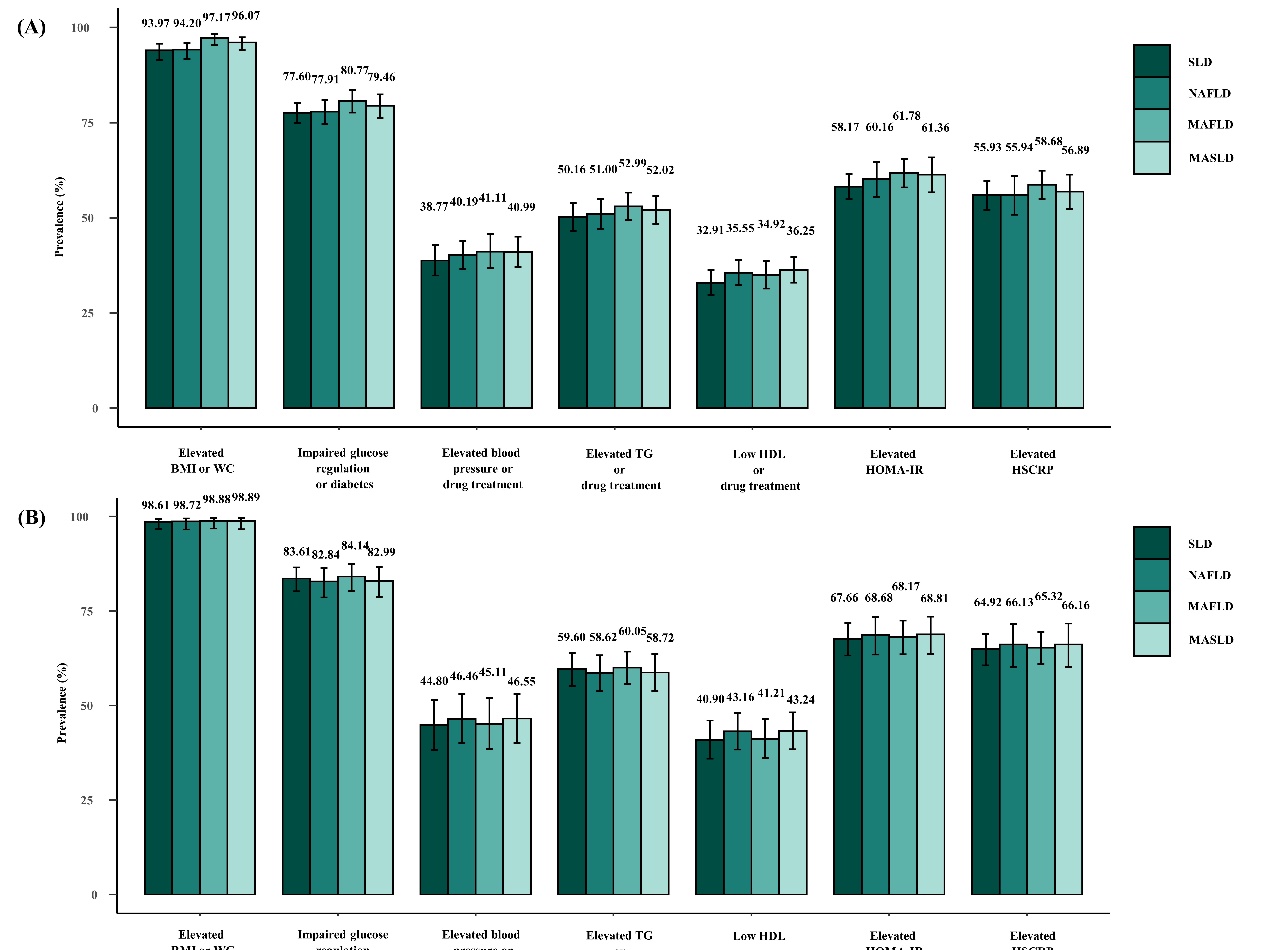


**Figure S5** Weighted prevalence of SLD (CAP ≥ 248 dB/m), NAFLD, MAFLD, and MASLD (A) and associated prevalence of ALF (B) among different number of CMRF.


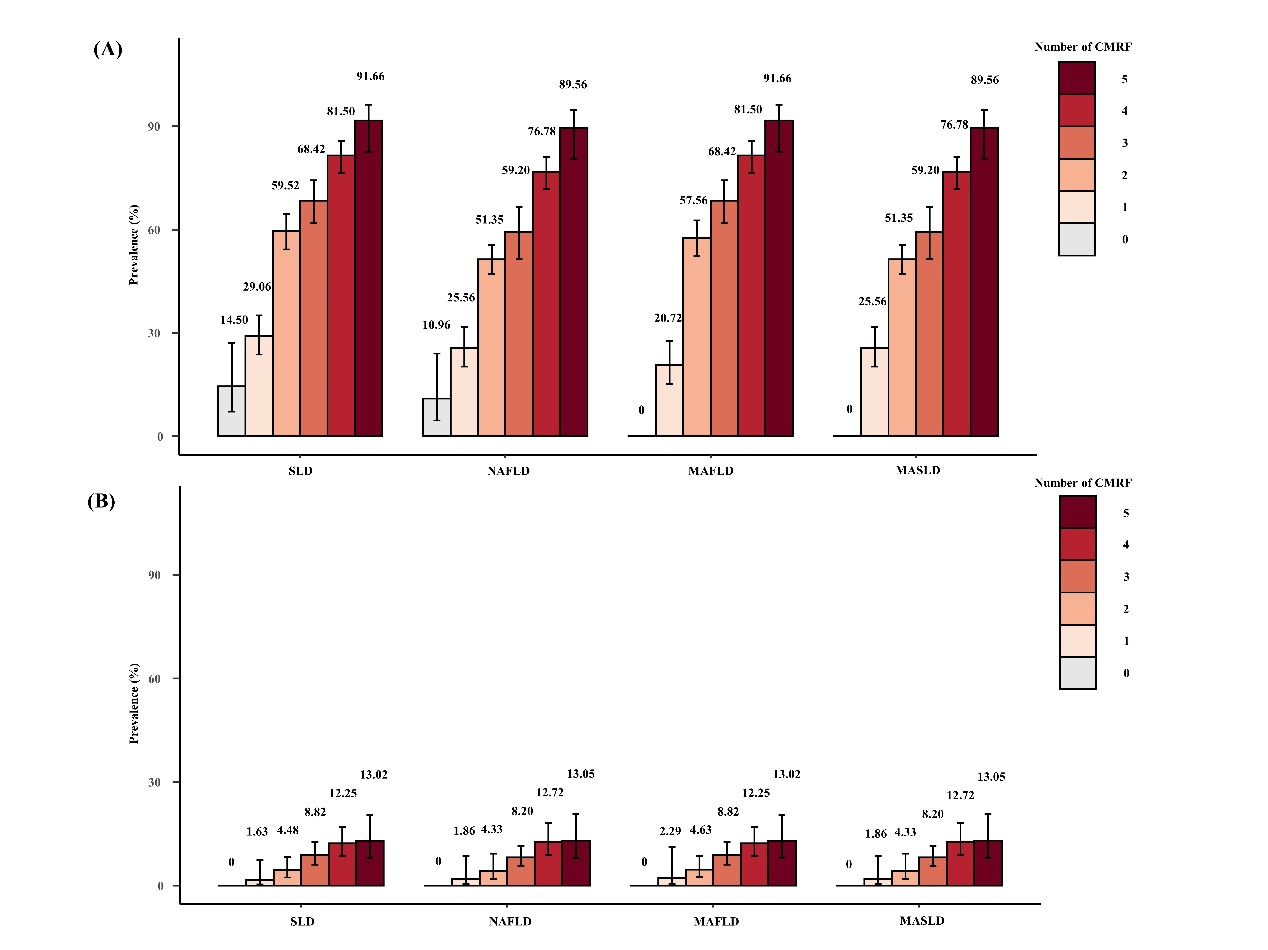


**Figure S6** Weighted prevalence of SLD (CAP ≥ 302 dB/m), NAFLD, MAFLD, and MASLD (A) and associated prevalence of ALF (B) among different number of CMRF.


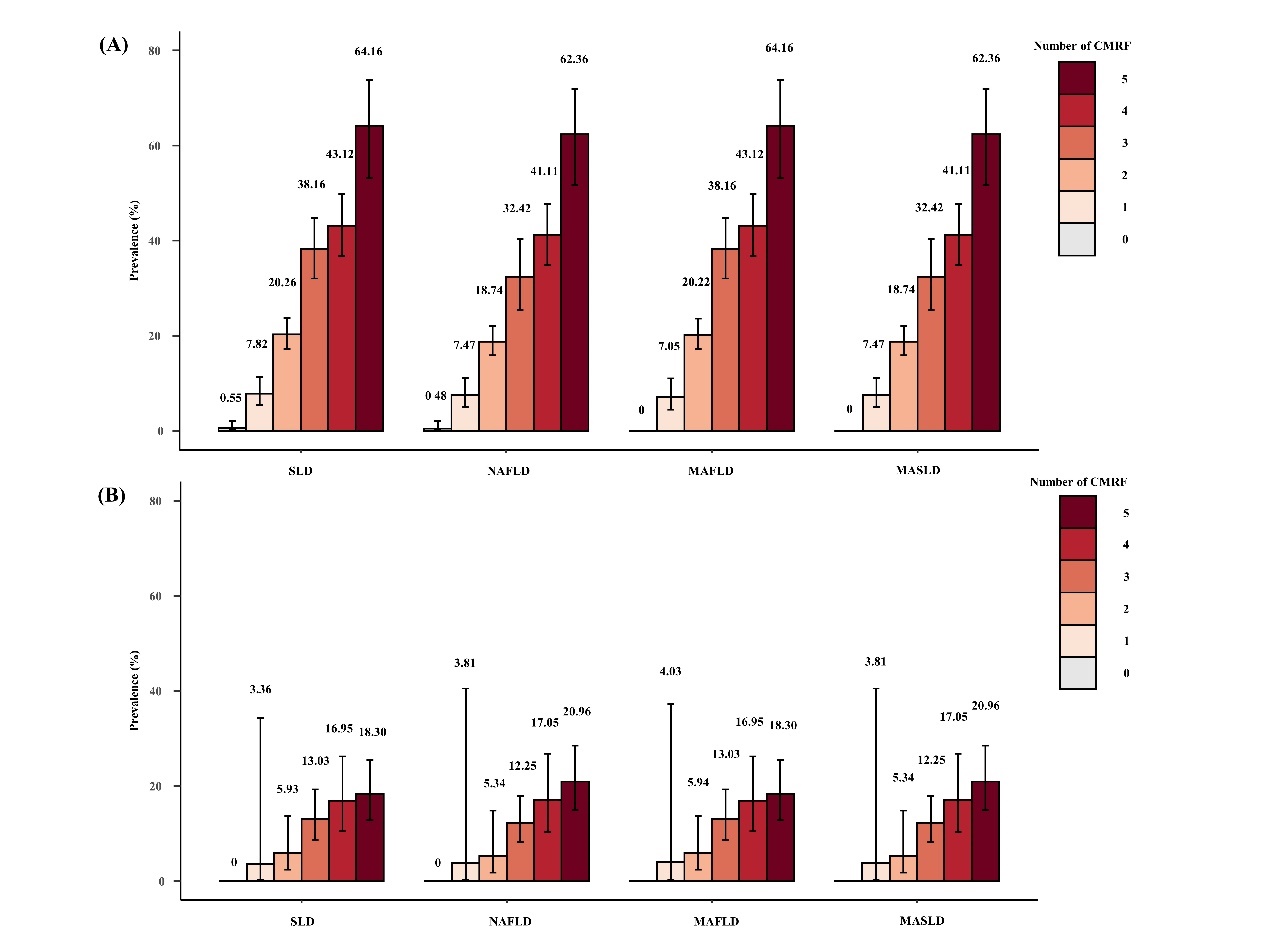


**Referrences**

1. Ruan Z, Lu T, Chen Y, Yuan M, Yu H, Liu R, Xie X: **Association Between Psoriasis and Nonalcoholic Fatty Liver Disease Among Outpatient US Adults**. *JAMA Dermatol* 2022, **158**(7):745-753.

2. **Alcohol Use Questionare** [<https://wwwn.cdc.gov/Nchs/Nhanes/2017-2018/P_ALQ.htm#ALQ121>]

3. **What Is A Standard Drink?** [<https://www.niaaa.nih.gov/alcohols-effects-health/overview-alcohol-consumption/what-standard-drink>]

4. Rinella ME, Lazarus JV, Ratziu V, Francque SM, Sanyal AJ, Kanwal F, Romero D, Abdelmalek MF, Anstee QM, Arab JP *et al*: **A multi-society Delphi consensus statement on new fatty liver disease nomenclature**. *J Hepatol* 2023.

5. **Classification and Diagnosis of Diabetes: Standards of Medical Care in Diabetes-2020**. *Diabetes Care* 2020, **43**(Suppl 1):S14-s31.

6. Williams B, Mancia G, Spiering W, Agabiti Rosei E, Azizi M, Burnier M, Clement DL, Coca A, de Simone G, Dominiczak A *et al*: **2018 ESC/ESH Guidelines for the management of arterial hypertension**. *European heart journal* 2018, **39**(33):3021-3104.

7. Younossi ZM, Stepanova M, Afendy M, Fang Y, Younossi Y, Mir H, Srishord M: **Changes in the prevalence of the most common causes of chronic liver diseases in the United States from 1988 to 2008**. *Clinical gastroenterology and hepatology : the official clinical practice journal of the American Gastroenterological Association* 2011, **9**(6):524-530.e521; quiz e560.

8. Eslam M, Newsome PN, Sarin SK, Anstee QM, Targher G, Romero-Gomez M, Zelber-Sagi S, Wai-Sun Wong V, Dufour J-F, Schattenberg JM *et al*: **A new definition for metabolic dysfunction-associated fatty liver disease: An international expert consensus statement**. *Journal of Hepatology* 2020, **73**(1):202-209.

9. Eslam M, Sanyal AJ, George J: **MAFLD: A Consensus-Driven Proposed Nomenclature for Metabolic Associated Fatty Liver Disease**. *Gastroenterology* 2020, **158**(7):1999-2014.e1991.

10. Eslam M, Sarin SK, Wong VW, Fan JG, Kawaguchi T, Ahn SH, Zheng MH, Shiha G, Yilmaz Y, Gani R *et al*: **The Asian Pacific Association for the Study of the Liver clinical practice guidelines for the diagnosis and management of metabolic associated fatty liver disease**. *Hepatol Int* 2020, **14**(6):889-919.
